# Application of Differential Network Enrichment Analysis for Deciphering Metabolic Alterations

**DOI:** 10.3390/metabo10120479

**Published:** 2020-11-24

**Authors:** Gayatri R. Iyer, Janis Wigginton, William Duren, Jennifer L. LaBarre, Marci Brandenburg, Charles Burant, George Michailidis, Alla Karnovsky

**Affiliations:** 1 Department of Computational Medicine and Bioinformatics, University of Michigan Medical School, Ann Arbor, MI 48109, USA; griyer@umich.edu (G.R.I.); wld@med.umich.edu (W.D.); mbradenb@umich.edu (M.B.); 2 Michigan Regional Comprehensive Metabolomics Resource Core, Biomedical Research Core Facilities, University of Michigan Medical School, Ann Arbor, MI 48109, USA; wiggie@med.umich.edu; 3 Department of Nutritional Sciences, University of Michigan School of Public Health, Ann Arbor, MI 48109, USA; jenlab@med.umich.edu; 4 Taubman Health Sciences Library, University of Michigan, Ann Arbor, MI 48109, USA; 5 Department of Internal Medicine, University of Michigan Medical School, Ann Arbor, MI 48109, USA; burantc@med.umich.edu; 6 Department of Statistics, University of Florida, Gainesville, FL 32611, USA

**Keywords:** partial correlation networks, differential networks, enrichment analysis, metabolic disorders, metabolomics and lipidomics

## Abstract

Modern analytical methods allow for the simultaneous detection of hundreds of metabolites, generating increasingly large and complex data sets. The analysis of metabolomics data is a multi-step process that involves data processing and normalization, followed by statistical analysis. One of the biggest challenges in metabolomics is linking alterations in metabolite levels to specific biological processes that are disrupted, contributing to the development of disease or reflecting the disease state. A common approach to accomplishing this goal involves pathway mapping and enrichment analysis, which assesses the relative importance of predefined metabolic pathways or other biological categories. However, traditional knowledge-based enrichment analysis has limitations when it comes to the analysis of metabolomics and lipidomics data. We present a Java-based, user-friendly bioinformatics tool named Filigree that provides a primarily data-driven alternative to the existing knowledge-based enrichment analysis methods. Filigree is based on our previously published differential network enrichment analysis (DNEA) methodology. To demonstrate the utility of the tool, we applied it to previously published studies analyzing the metabolome in the context of metabolic disorders (type 1 and 2 diabetes) and the maternal and infant lipidome during pregnancy.

## 1. Introduction

Over the last decade, the field of metabolomics has become an integral part of basic, clinical, and translational research. The metabolome provides a readout of the underlying cellular and biochemical events that reflect individual genetic makeup [1], epigenetics [2], the microbiome [3], and environmental exposures, including diet [4,5]. Metabolic profiling has been successfully applied to biomarker discovery and the assessment of disease risk and progression in cancer [6,7], cardiovascular [8,9] and renal diseases [10,11], and type 1 (T1D) [12,13] and type 2 diabetes (T2D) [14,15].

Metabolism is interconnected through several major metabolic hubs, e.g., glucose-6-phosphate, pyruvate, acetyl-CoA, and malonyl-CoA. Beyond these central nodes, metabolic pathways have secondary rate-limiting steps that are often controlled by metabolites affecting multiple pathways (such as AMP, citrate, NAD, etc.), as well as by post-translational modifications of proteins regulating the pathway. Evaluating changes in the connectivity of the metabolome could help to understand how these pathways are affected in physiological and disease states.

Experimental design in metabolomics commonly involves assessment of metabolite levels in two or more disease conditions or experimental groups. Metabolomics data acquired from such experiments are amenable to univariate analysis, followed by pathway mapping and enrichment analysis. Enrichment analysis, originally developed for gene expression data, reduces data involving hundreds of altered genes or metabolites to smaller and more interpretable sets of altered biological ‘concepts’, helping generate testable hypotheses. The most common types of enrichment analysis are variants of over-representation analysis (ORA) or set enrichment analysis (SEA) [16]. In both cases, statistical tests are performed to assess the enrichment or depletion of a set of metabolites in a specific pathway against a background or reference set [17].

Several bioinformatics tools implementing the above data analysis workflow for metabolomics have been developed [18,19]. While overall this approach has proven to be extremely useful, each of the individual methods involved has limitations. First, univariate analysis considers only individual metabolites and does not account for the interactions between them. Indeed, biological constraints on metabolism result in many metabolites being highly correlated in biological samples (for instance, branched chain amino acids). Second, the application of metabolite pathway mapping and enrichment analysis is hampered by the low coverage of experimentally determined metabolites in biological pathway databases [20]. This is particularly true for lipids and secondary metabolites. The low coverage can in part be explained by the differences between chemistry-centric metabolomics experiments and genome-centric pathway databases. This problem is further compounded by the relatively small number of known metabolites measured in most experiments which limits both the statistical significance and overall reliability of analyses.

We present a user-friendly tool, Filigree, that overcomes many of the limitations of existing methods. Filigree implements our recently published differential network enrichment analysis (DNEA) method [21]. DNEA provides an alternative to traditional pathway-centric approaches by leveraging the underlying structure of the data and inferring associations among metabolites directly from experimental measurements. These associations can be quantified by partial correlations that measure the conditional dependence between metabolites, thus allowing elimination of spurious, non-informative associations. In lieu of predefined pathways, DNEA generates stable subnetworks comprised of biochemically and structurally related metabolites. It accounts for both changes in network structure and the differential abundance of metabolites when assessing significance of subnetworks, thus providing a systems level view of the data. To demonstrate the utility of Filigree, we applied it to previously published studies assessing the metabolome in the context of metabolic disorders (T1D and T2D) and the maternal and infant lipidome during pregnancy. Filigree is freely available at http://metscape.ncibi.org/filigree.html. 

## 2. Results and Discussion

**Figure 1:**
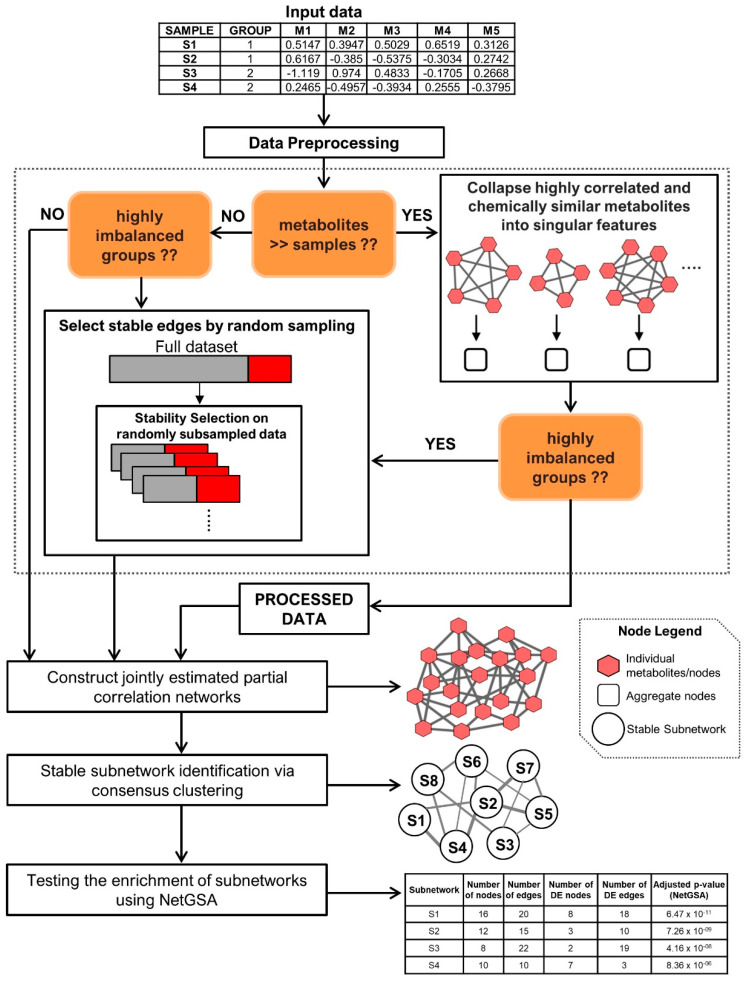
Schematic representation the data analysis pipeline.

The DNEA method [21] implemented in Filigree includes three main steps: (1) joint estimation of the partial correlation network (PCN) across two groups of samples, (2) unsupervised clustering of the resulting PCN using consensus clustering to obtain densely connected subnetworks, and 3) testing the subnetworks for enrichment using the NetGSA algorithm (Figure 1) [22,23]. As mentioned in [21], the groups can correspond to treatment-control conditions, disease subtypes, etc. Further details of the DNEA algorithm are described in Supplementary Methods. Figure 1 depicts our analysis pipeline and describes the Filigree/DNEA workflow.

### 2.1. DNEA Analysis Reveals Dysregulation of Metabolite Networks in T1D vs. Non-Diabetic Mice

**Figure 2:**
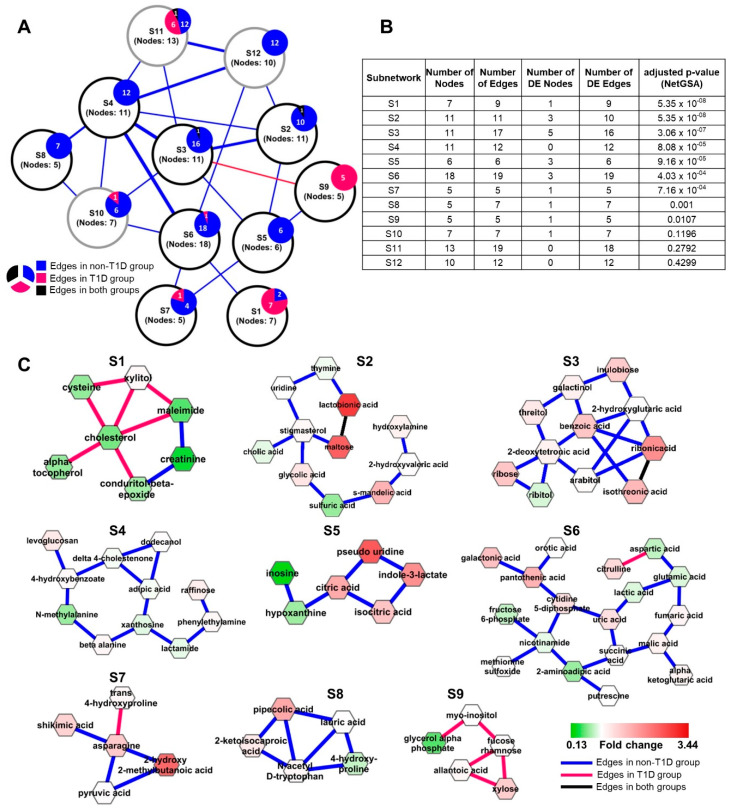
(**A**) Overview of T1D mouse model Filigree network showing associations between all the subnetworks. Each node represents a subnetwork with the overlaying pie charts showing the distribution of the intra-subnetwork edges. Inter-subnetwork edges are weighted by the total number of edges. Nodes with black outline are significantly differential by NetGSA (**B**) NetGSA output from Filigree showing subnetwork information and statistics. (**C**) Significantly differential subnetworks. Nodes are colored based on fold change (T1D over non-T1D).

We utilized Filigree to perform DNEA analysis of the metabolomics data from NOD mice that either progressed or did not progress to overt T1D [24,25]. Plasma metabolites from T1D and non-diabetic NOD mice produced a PCN with stronger connectivity in the non-diabetic mice (Figure 2A). The subsequent analysis steps identified twelve stable subnetworks within the resulting PCN (Supplementary Figure S1). Nine of the these were significantly differential between T1D and non-diabetic mice (FDR < 0.05) (Figure 2B,C).

Seven out of nine differential subnetworks contained edges present in non-diabetic mice that were disrupted in diabetic animals. Four out of these, S2, S3, S4, and S6, are highly interconnected. These subnetworks contain nucleobases, ribose and its reduction products, nucleic acids, amino acids, and also several sugars and sugar-related metabolites. (see Supplementary Table S1 for the complete list of metabolic pathways). We note that several edges connecting metabolites in these subnetworks represent oxidation/reduction reactions. For instance, galactinol, a sugar alcohol, is the reduction product of galactose and ribitol is a reduction product of ribose. This suggests that the connectivity between metabolites in these subnetworks is disrupted due to changes in redox potential that accompany the progression to T1D. Thus, a general decrease in the redox state of cells may contribute to the changes in the connectivity of metabolites seen in the plasma in T1D.

The association between the cellular redox state and the metabolome is further supported by S1 and S9, which contain predominantly diabetic edges (Figure 2C). In both subnetworks, the enrichment is driven primarily by the differential edges, while most metabolites (nodes) are not significantly differentially expressed and therefore would not be prioritized by univariate analysis (Figure 2B).

S1 consists of metabolites either directly or indirectly related to increased oxidative stress. Oxidative stress is a widely accepted complication accompanying the pathogenesis of diabetes by way of increased free radical (ROS) concentrations caused by hyperglycemia as well as decreased levels of major antioxidants such as glutathione [26], leading to significant damage to pancreatic islet beta cells responsible for insulin secretion [27]. Glutathione (gamma-glutamyl-cysteinyl-glycine) is a highly abundant tripeptide in the human body known to play a vital role in defense against oxidative stress as a free radical scavenger [28]. The bulk of the blood glutathione is found within erythrocytes (millimolar concentrations) while levels in the plasma tend to be in the micromolar range. Diminished levels of blood glutathione have been implicated both in T1D and in T2D [29,30,31,32]. While glutathione was not measured in this experiment, we speculate that reduced level of this metabolite can influence the levels of several S1 metabolites, including cysteine, cholesterol, creatinine, and xylitol. Cysteine, one of the three amino acid constituents of glutathione, is present in this subnetwork with lower levels in diabetic mice. It has been postulated that reduced levels of glutathione in type 1 diabetes is a consequence of increased utilization rather than decreased synthesis, thus resulting in reduced levels of cysteine [31]. A hub node of S1 is cholesterol. Counterintuitively, we see decreased levels of cholesterol in diabetic mice. This is likely due to the inhibitory effect of diminished glutathione on the enzyme HMG-CoA reductase, the rate-controlling enzyme in the cholesterol synthesis pathway (Malveonate pathway). Glutathione has been suggested to be one of the key activators of HMG-CoA reductase by maintaining the enzyme in its active, reduced sulfahydryl state [33,34,35,36]. Moreover, insulin has also been shown to be an activator of HMG-CoA reductase in a mechanism similar to glutathione [37]. Depleted glutathione also has an inhibitory effect on the enzyme creatine kinase (CK), responsible for the phosphorylation of creatine to phoshpocreatine, likely due to thiol oxidation of the sulfahydryl groups of the enzyme [38,39]. A reduction in CK activity leads to a decrease in phosphocreatine levels which further causes a decrease in creatinine levels, a product of phosphocreatine utilization. Consequently, we observe creatinine in subnetwork S1 at lower levels in diabetic mice. Additionally, xylitol, a five-carbon sugar alcohol and widely used sugar-substitute, has also been shown to serve as a glutathione-reducing compound in vitro and in vivo [40,41]. While we did not see a significant difference in the levels of xylitol between diabetic and non-diabetic mice, its potential association with glutathione is a possible reason for its presence in subnetwork S1. Finally, we see alpha-tocopherol (Vitamin E) in subnetwork S1. This is not unexpected as alpha-tocopherol is a well-known potent antioxidant, similar to glutathione. It is therefore not surprising that we see lower levels of alpha-tocopherol in diabetic mice.

Several S1 metabolites are exogenous compounds often measured in plasma and urine. In general, these compounds are decreased in T1D mice and also have differential connectivity, suggesting that their metabolism is disrupted in T1D. Alternatively, exogenous compounds may not be easily absorbed in the intestine in T1D, potentially due to altered intestinal permeability. In T1D, there are marked changes in the intestinal morphology and expression of transporters [42] and increased intestinal permeability [43], altering the entry of exogenous substances with additional effects on cellular metabolism. These findings also support previously described disruptions in metabolism associated with T1D, including alterations in mitochondrial metabolism, increased oxidative stress, and changes in redox state [44]. Indeed, Fahrmann et al. [24] previously reported increased levels of sugar-related metabolites, branched chain amino acids, gluconic acid and nitric oxide-derived saccharic acid markers of oxidative stress in T1D mice. Our network-based approach confirms and extends the understanding of alteration in metabolism that occurs in T1D, including changes in the metabolism of nucleotides (S2–S5). Because these alterations are found in plasma, the tissue-specific origins of disruption in metabolism cannot be precisely localized.

### 2.2. Connectivity of Metabolite Networks Differs between Non-Diabetics and Individuals Who Later Developed T2D from the Framingham Heart Study (FHS) Offspring Cohort

The FHS Offspring Cohort has been studied extensively and biomarkers for risk of cardiovascular disease and T2D have been identified [14,45]. We used DNEA to examine metabolomics data from 100 FHS subjects who developed T2D over the course of the subsequent twenty years (T2D-prone) and 674 subjects who remained non-diabetic (T2D-free). This highly imbalanced group distribution makes it difficult to recover robust and stable PCNs [46]. Statistical theory [47] suggests that subsampling approaches can reduce the bias towards the group with higher number of samples. We created a subsampling approach that allows a stable network topology to be obtained and reduces the number of edges in the non-diabetic group (described in Methods). The number of edges recovered with and without subsampling, within each group, is reported in Table 1.

**Figure 3:**
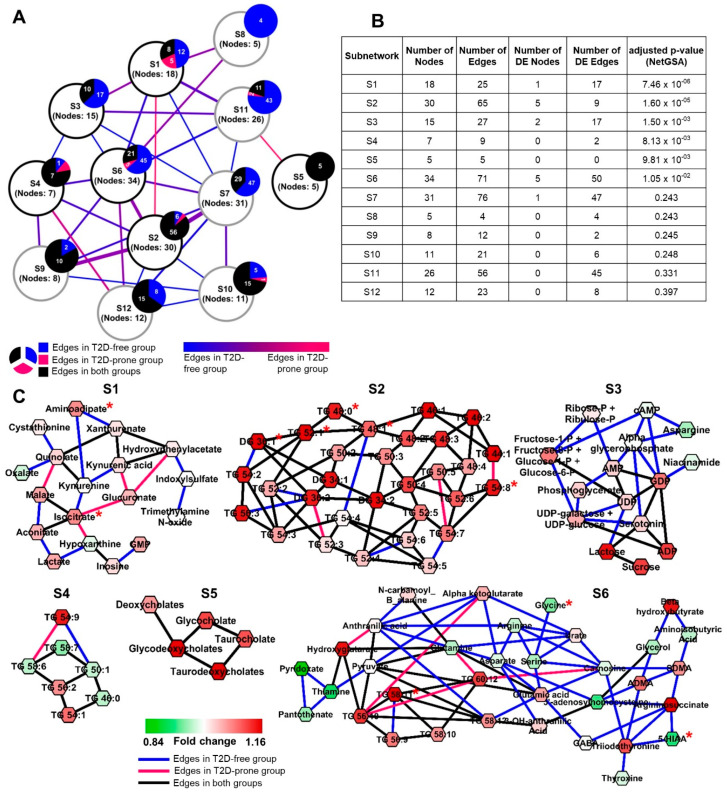
(**A**) Overview of the Framingham Heart Study Offspring Cohort T2D network showing associations between all the subnetworks. Each node represents a subnetwork with the overlaying pie charts showing the distribution of the intra-subnetwork edges. Inter-subnetwork edges are weighted by the total number of edges. Nodes with black outline are significantly differential by NetGSA. (**B**) NetGSA output showing subnetwork information and statistics. (**C**) Significantly differential subnetworks. Nodes are colored based on fold change (T2D-prone over T2D-free). Nodes marked with red asterisk (*) have been reported as T2D predictors by Merino and colleagues (2018).

Our analysis identified substantial network differences between T2D-prone and T2D-free groups (Figure 3A). The algorithm identified twelve stable subnetworks (Supplementary Figure S2) within the resulting PCN, with six subnetworks significantly differing between T2D-prone and T2D-free groups (FDR < 0.05) (Figure 3B,C). Similar to our findings in T1D, there were fewer edges in T2D-prone compared to T2D-free networks (Supplementary Figure S3). This tendency is especially apparent in subnetworks S1, S3, and S6 (Figure 3B).

The most significant subnetwork (S1) includes intermediates of tryptophan, cysteine, lysine, tyrosine, and phenylalanine metabolism (Supplementary Table S2 and Figure S4). Dysregulation of tryptophan metabolism [48,49] and elevated level of 2-amnionadipic acid have been associated with the development of T2D [50]. Previous studies in the FHS Offspring Cohort found that branched chain and aromatic amino acids were positively associated with the risk of developing T2D [14]. The subnetwork containing branched chain amino acids (S11) is not significantly differential between groups (Supplementary Figure S5), consistent with the findings of Merino and colleagues [51] who found that branched chain amino acids (BCAAs) were not predictive of T2D in this sample cohort, perhaps due to the relatively small differences in insulin resistance between the T2D-prone and T2D-free individuals in these data. Subnetwork S1 also includes several intermediates of purine metabolism (Supplementary Table S2). Increased levels of uric acid, the end-product of purine metabolism, is a common finding in obese T2D patients and has been implicated in the pathogenesis of metabolic syndrome disorders [52,53]. These latter studies suggest the role of hyperuricemia in increased mitochondrial oxidative stress. While uric acid was not measured in the FHS Offspring Cohort study, increases in GMP and hypoxanthine may reflect the upstream hyperuricemia in the T2D-prone subjects. Additionally, subnetwork S1 includes the TCA cycle metabolites malate, isocitrate and aconitate, which are all increased in T2D-prone subjects, suggesting alterations in mitochondrial metabolism.

Subnetwork S3 contains a higher proportion of edges in the non-diabetic group and is populated by sugars and sugar phosphates in the glycolysis and pentose shunt pathways, nucleotides, and sugar nucleotides. T2D-prone subjects have higher plasma levels of these sugars and sugar-derivatives than non-diabetic subjects. Taken together, the metabolite alterations seen in subnetworks S1 and S3 are indicative of widespread changes in the orderly flux of metabolites through mitochondria in diabetes-prone individuals. While not a new concept (reviewed in [54]), our results demonstrate the utility of the DNEA approach to provide insights into altered whole body metabolism using plasma metabolomics.

Subnetworks S2 and S4 were statistically significant in our analysis, even though the majority of edges are non-differential. These subnetworks are primarily made up of long-chain (C44-C58) polyunsaturated triglycerides (PUFA-TGs) with the additional inclusion of four diglyceride (DG) species (DG 34:1, DG 34:2, DG 36:1, DG 36:2), two saturated triglycerides (TG 46:0 and TG 48:0) and six monounsaturated triglycerides (TG 44:1, TG 46:1, TG 48:1, TG 50:1, TG 52:1, and TG 54:1). Most TG lipids, except TG 46:0, TG 50:1, TG 58:6, and TG 58:7, are present at higher levels in T2D-prone subjects. Overall, the enrichment of these two subnetworks is primarily driven by differential expression of the nodes. Increased plasma triglycerides have been reported as an independent predictor of T2D in several prospective cohort studies [55,56]. Additionally, triglycerides tend to be highly correlated with each other and typically form densely connected clusters in correlation networks [21]. The presence of a separate smaller triglyceride subnetwork (S4) may be due to the absence in the dataset of key triglyceride species that could link these subnetworks.

Subnetwork S5 exclusively contains bile acids with non-differential edges, suggesting that the differences between T2D-prone and T2D-free subjects in this case are driven by differential expression of the metabolites. Bile acids are the primary route of cholesterol catabolism and are synthesized by the oxidation of the latter by the action of the rate-limiting enzyme cholesterol 7 alpha-hydroxylase. Alterations in bile acid metabolism have been associated with T2D [57,58,59,60,61]. Additionally, obese T2D individuals have increased fasting and post-prandial total bile acid concentrations, due to increased enterohepatic circulation [62].

Subnetwork S6 contains several amino acids and their derivatives, TCA cycle intermediates, vitamin B metabolites and thyroid hormones (Supplementary Table S2). In general, network connectivity was higher in the T2D-free group compared to the T2D-prone group. The levels of the individual amino acids and primary metabolites in this subnetwork are generally lower in T2D-prone group. Reductions in glycine and glutamine-to-glutamate ratio have been found in T2D subjects and in T2D-prone individuals [63]. The basis for the changes in arginine and aspartate levels, which are reduced in concert with other amino acids (save glutamate) in this network are less clear. We did not observe differential connectivity among the polyunsaturated fatty acid-containing triglycerides (PUFA-TGs). However, their levels were increased in the T2D-prone group, consistent with the overall increase in the TGs in the T2D-prone population (Supplementary Table S2).

Our analysis of the FHS Offspring Cohort metabolomics data supports many of the previous findings elucidating the role of changes in amino acid metabolism and increased oxidative stress in the prediction of T2D onset. Additionally, of the nineteen metabolites prioritized by Merino and colleagues (from the same dataset) that significantly improved T2D prediction in a model including traditional T2D risk factors [51], ten were part of our significantly differential subnetworks S1–S6 (Supplementary Table S2). With these previously observed metabolite relationships as a foundation, our subnetworks can provide further biochemical context and help build on the understanding of metabolic changes that eventually lead to disease.

### 2.3. Subnetworks of Lipids Relate to Infant Birth Weight in the Michigan Mother-Infant Pairs (MMIP) Cohort

**Figure 4:**
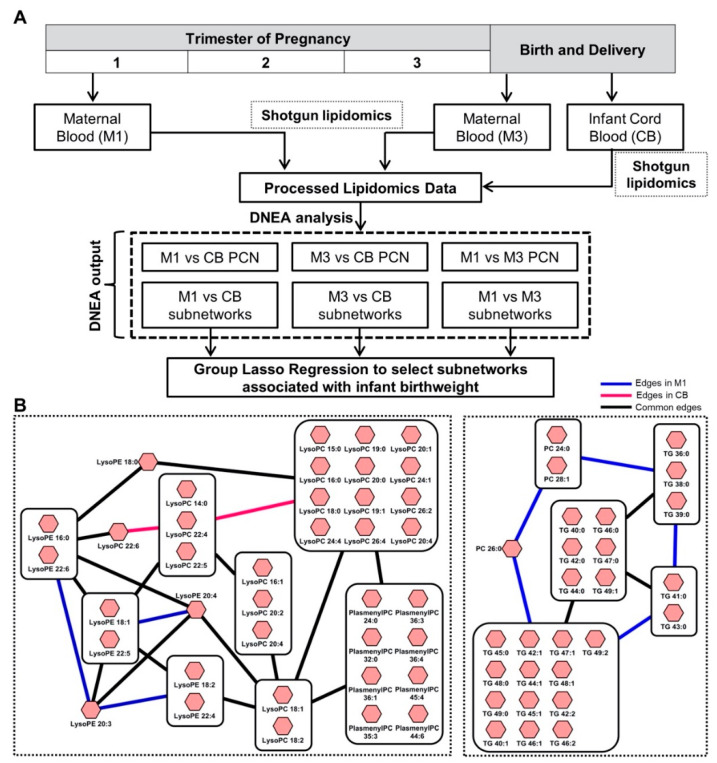
(**A**) Michigan Mother-Infant Pairs (MMIP) study design. 106 pregnant women were monitored through the course of their pregnancy. Maternal plasma samples were collected at the first trimester (M1) and at time of delivery (M3), along with Cord Blood (CB). Data from subsequent lipidomics experiments was analyzed in a pairwise manner using Filigree and resulting subnetworks were tested for their association with infant birth weight in a group lasso regression model. (**B**) Top two M1 vs. CB subnetworks strongly associated with infant birth weight. LPC-LPE-PlasmenylPC subnetwork in infant Cord Blood and PC-TG subnetwork during the first trimester of the mother are strongly associated with infant birth weight. Large square nodes containing smaller nodes within them represent ‘aggregated’ nodes with their individual lipid species.

We used Filigree to analyze the MMIP dataset [64], comparing the lipidomes of women at different stages of pregnancy and their offspring (Figure 4A). Capitalizing on the method’s ability to identify functionally related metabolic modules, we sought to explore the association of subnetworks with infant birth weight (BW). Accordingly, we performed three pairwise comparisons (M1 vs. M3, M1 vs. CB, and M3 vs. CB). Since the dataset contained 670 lipids and 106 samples, we used the feature aggregation functionality of the tool (described in Methods) to reduce the dimensionality of the data. Table 2 gives the reduced feature count for each of the comparisons and the percent of feature reduction. Overall, a 55–60% reduction was chosen, yielding feature counts comparable to the sample size. Filigree results are summarized in Table 2. Most of the identified subnetworks were significantly enriched in each of the pairwise comparisons: 14/19 in M1, 19/20 in M3, and 9/12 in CB (Table 2). Summary statistics for each of these subnetworks is detailed in Supplementary Table S3. Consistent with our previous observations [21], lipids from the same or highly related classes were often found within the same subnetworks, such as diglycerides (DG) and triglycerides (TG), phosphatidylcholines (PC) and phosphatidylethanolamines (PE), and lysophosphatidylcholines (LPC) and lysophasphatidylethanolamines (LPE) (Figure 4B). Most subnetworks included differential edges at each time point, indicating changes in the connectivity of the lipidome during pregnancy.

Next, we assessed whether any of the identified subnetworks were associated with infant BW, which is of particular interest due to its relationship with future weight gain and risk for metabolic disease [65]. We used group lasso regression [66] (described in Methods) to model our Filigree subnetworks as predictors and Fenton BW [67] (BW normalized for gestation period and sex) as the outcome variable. In the M1 vs. CB comparison, two subnetworks containing LPC-LPE-PlasmenylPC (S18) and PC-TG (S12) components displayed strong association with BW (Figure 3B). The LPC-LPE-PlasmenylPC subnetwork, composed of lipids with saturated, monounsaturated, and polyunsaturated fatty acid tails, showed a stronger association with BW in CB. Previous work has emphasized the relationship between CB LPCs and BW [64,68], but no previous studies have reported an association with PlasmenylPCs. Plasmalogen formation is primarily regulated by peroxisomes and it has been proposed that plasmologens are related to inflammation and oxidative stress [69], potentially explaining their association with BW. The PC-TG subnetwork displayed a stronger association with BW in M1. This network is composed of lipids that contain saturated fatty acid tails with 12–16 carbons. Our results expand on the previous analysis [64] that found minimal associations between the M1 lipidome and BW, emphasizing the advantage of our network-based approach. We hypothesize that lipids with saturated fatty acids play a role in establishing BW in the first trimester of pregnancy (8–14 weeks), highlighting the plasticity of the developing fetus in early gestation, responding potentially through epigenetic modifications [70]. Interestingly, the edges within the subnetwork diminish in CB, suggesting different connectivity between these saturated lipids at each time point, potentially due to changes in insulin sensitivity during pregnancy [71].

In the M3 vs. CB comparison, two subnetworks containing LPC-LPE (S6) and PC-PlasmenylPC-PlasmenylPE-DG-TG (S10) components displayed strong associations with BW (Supplementary Figure S6 and Table S4). These subnetworks were associated with BW specifically in the CB, rather than maternal plasma (M3). The LPC-LPE subnetwork only includes one PlasmenylPC (PlasmenylPC 26:0), suggesting that plasmalogens are less strongly correlated with lysophospholipids in this comparison. Almost a complete overlap of lysophospholipids was observed between M1-CB S18 and M3-CB S6. The PC-PlasmenylPC-PlasmenylPE-DG-TG subnetwork contains lipids with long-chain and very long-chain polyunsaturated fatty acid tails. Previous work [64] has suggested the association between BW and CB polyunsaturated TGs and DGs. However, our approach additionally shows the interconnectivity between multiple lipid classes. Since polyunsaturated fatty acids are preferentially transferred from maternal to fetal circulation [72], our results may suggest a mechanism that modifies fetal growth and BW for optimal development. Previous studies using polyunsaturated fatty acid supplementation during pregnancy have yielded mixed results [73], warranting further analyses of the interconnectivity of these lipid classes and their relationship to BW.

In the M1 vs. M3 comparison, two subnetworks containing DG-TG (S7) and LPC-LPE (S14) components displayed strong associations with infant BW, led by maternal blood (M3) (Supplementary Figure S7 and Table S5). The LPC-LPE subnetwork contains the same lysophospholipids as M1-CB S18 and M3-CB S6. These results suggest that maternal late gestation lysophospholipids are related to BW, potentially due to the active transport of lysophospholipids from maternal plasma to the CB by the major facilitator superfamily domain containing 2a (MFSD2a) protein [74]. Thus, enriched subnetworks obtained from the Filigree have meaningful biological significance and can be utilized to advance lipidomics data analysis by looking at their association with other phenotypes of interest.

In conclusion, we presented a novel bioinformatics approach for gaining new insights into high dimensional metabolomics data as implemented in our tool, Filigree. Our method helps overcome common challenges of pathway-based enrichment testing approaches, providing robust results even with limited sample sizes and highly imbalanced experimental group designs. 

To the best of our knowledge, currently there is no other tool with comparable analysis pipeline. However, there are many tools for computing partial correlation networks and performing traditional pathway-based enrichment analysis. We used several of these methodologies to analyze the T1D dataset (see Supplementary Results). While partial correlation networks can be built with existing methodologies [75], Filigree provided a clear advantage in network estimation. In the T1D dataset, the number of metabolites far exceeded the number of samples, considerably restricting the number of statistically significant edges that could be recovered by other existing methods [75]. Our analysis also demonstrated that topology-based enrichment method implemented in Filigree is more powerful than traditional enrichment testing because it has the ability to provide information about changes in topology across the biological conditions.

In re-analyzing several existing datasets with Filigree, we observed a strong differential connectivity in metabolite networks in T1D and T2D and were also able to demonstrate various associations with infant BW in the lipidomes of pregnant women. Filigree is particularly useful as a hypothesis-generating tool. The results presented here suggest potential follow-up studies that could shed light on additional metabolic factors contributing to T1D and T2D and on potential lipidomic influences on BW during pregnancy.

## 3. Materials and Methods 

### 3.1. Filigree Application

The input to the tool is a plain text file containing per-sample unadjusted intensity values and group information. The output consists of three. csv files: (1) an ‘edgelist’ containing metabolite pairs and partial correlation values between them; (2) a ‘nodelist’ containing information about the differential status of each metabolite, along with its statistical significance and subnetwork membership, and (3) a NetGSA results file containing information about subnetworks, including number of edges/nodes and statistical significance of each subnetwork. These files can be easily imported into network visualization software such as Cytoscape for further exploration [76]. Additionally, the user can browse the interactive HTML files automatically generated by Filigree. 

### 3.2. Extensions of DNEA Methodology

The DNEA method works particularly well both theoretically [46] and empirically [21] when group sizes are fairly balanced, and the number of metabolites is a low multiple of the sample size. However, in many applications the two groups under consideration may be grossly imbalanced or the number of samples severely limited. To that end, we developed several extensions to the DNEA methodology (described below) that improve its versatility, including (i) feature aggregation and (ii) group subsampling to attain more balanced sample sizes across groups.

#### 3.2.1. Feature Aggregation

Since network density and stability are strongly dependent on the ratio of features (metabolites and/or lipids) to samples [46], a preprocessing step to aggregate highly similar or redundant features may be appropriate. This step helps reduce the dimensionality of the data to promote the retrieval of more interpretable PCNs and is therefore highly recommended for datasets where the number of features is a high multiple of the number of samples. 

We implemented an optional data preprocessing step for aggregation of highly similar or redundant features in the dataset in order to recover more stable PCNs. Feature aggregation performs optimally when data are log-transformed, but not auto-scaled. Several types of aggregation are possible: (1) a purely data-driven approach that collapses features with highly similar (Pearson) correlation profiles into singular features, (2) a purely knowledge-driven method that collapses chemically similar metabolites/lipids, or (3) a hybrid feature aggregation that collapses only features identified as chemically similar that also share a highly similar Pearson correlation profile. For options (2) or (3), the user may provide their own knowledge-based feature grouping file or can utilize the grouping file based on chemical similarities found in KEGG [77], HMDB [78] or LipidBlast [79]. For options (1) or (3), the user has the choice to view the features-to-sample-size ratio at various feature-aggregation tolerance values based on the correlation structure of the data. The user can then decide the extent of feature aggregation they wish to perform or can proceed with the recommended values. The output of this stage is a new data matrix where metabolites/lipids belonging to the same feature group are represented as singular features by computing their median intensity across all samples. The format of the new data matrix will be identical to that of the original input matrix.

#### 3.2.2. Group Subsampling

Highly imbalanced sample group sizes can result in PCNs where the smaller group is much sparser than the larger group, thus hindering interpretability of results. To address this issue, we modified the algorithm by using subsampling to create more balanced sample groups, leading to more stable and interpretable PCNs. The modified procedure is comprised of the following steps:

(1) Determine size of smaller group (*n_min_*);
(2) At every iteration of the stability selection (default value set to 500 iterations), create new data matrices for the two groups as follows:
  (a) For the larger group, randomly sample *alpha* × *n_min_* samples without replacement.
  (b) For the smaller group, randomly sample *beta* × *n_min_* samples without replacement. Additionally, in order to maintain some degree of randomness in the smaller group, (1 − *beta*) × *n_min_* samples are randomly chosen from this and added back.
(3) Fit the training model for the new subsamples of the data at every iteration;
(4) Obtain edge selection probabilities and retain edges with a selection probability of > *tau*;
(5) Use the selection probabilities as weights when estimating the partial correlation networks. Based on extensive experimentation, we recommend *alpha* = 1.3, *beta* = 0.9 and *tau* = 0.9, but the practitioner can also experiment with other values.

### 3.3. Datasets

#### 3.3.1. Mouse Model of T1D 

Previous studies [24,25] have generated and examined GC-MS metabolomics data from non-obese diabetic (NOD) mice, some of which progressed to overt T1D (chronic hyperglycemia) while others avoided progression (normoglycemia). Metabolomics data containing 163 named metabolites from 71 mice (30 diabetic and 41 non-diabetic) were downloaded from the Metabolomics Workbench (Study ST000057). Age- and sex-adjusted data [25] were log-transformed and autoscaled to have zero mean and unit variance.

#### 3.3.2. Framingham Heart Study (FHS) Offspring Cohort

The FHS Offspring Cohort is a longitudinal, community-based cohort that includes 3799 participants, aged 40–65 years, at the fifth quadrennial examination cycle 1991–1995 (baseline for our purposes) [80]. We downloaded plasma metabolite profiles (LC-MS/MS) for 956 subjects at baseline from the dbGaP database (https://www.ncbi.nlm.nih.gov/gap/). Approximately 10 years after the metabolomics analyses (2001–2005), subjects were re-recruited to be assessed for development of T2D, determined based on the following criteria: (1) fasting glucose ≥ 7 mmol/L, (2) 2-h glucose ≥ 11 mmol/L, and (3) consumption of oral hypoglycemics or insulin [51]. 674 subjects remained healthy while 100 subjects developed T2D (182 subjects had missing data in at least one of the variables). Age- and sex-adjusted data were log-transformed and autoscaled to have zero mean and unit variance.

#### 3.3.3. Michigan Mother-Infant Pairs (MMIP) Cohort

The Michigan Mother-Infant Pairs (MMIP) cohort [64] evaluated the plasma lipidome in 106 pregnant women during the first trimester (M1), at the time of delivery (M3), and within infant umbilical cord blood (CB). Comprehensive lipidomics profiling identified 670 lipid species from 17 different classes. Filigree was used to perform pairwise analyses between: (i) M1 vs. M3; (ii) M1 vs. CB, and (iii) M3 vs. CB, classifying differences in the connectivity of subnetworks between time points. We used the feature aggregation functionality of Filigree to collapse highly correlated and chemically similar lipids into singular features, making the feature space comparable to the sample size.

### 3.4. Group Lasso Regression

Filigree subnetworks generated from pairwise comparisons (M1 vs. CB, M3 vs. CB and M1 vs. M3) were tested for their association with infant birth weight (BW) at individual time points (M1, M3 and CB) in a group lasso regression [66] model using the R package gglasso [81]. Group lasso is an extension of the traditional lasso regression methodology [82] that incorporates prior information about the grouping of variables. In contrast to lasso regression, variable selection is performed on an entire set of variables (or predictors) instead of individual variables. Let y be a vector of length N and X be an N ×p matrix of features. Let the p features (or predictors) be divided into L groups such that there are $p_{l}$ predictors in group l. The matrix $X_{l}$ therefore represents predictors from the $l^{\mathrm{th}}$ group with a coefficient vector $\beta_{l}$. β estimates are obtained by solving the optimization problem,

(1) $$\underset{\beta \in R^{p}}{\min}\left({\left||y-\;\overset{L}{\underset{l=1}{\sum }}X_{l}\beta_{l}|\right|}_{2}^{2}+\;\lambda \overset{L}{\underset{l=1}{\sum }}\sqrt{p_{l}}{\left||\beta_{l}|\right|}_{2}\right)$$

Here, ${\left||\cdot |\right|}_{2}$ denotes the Euclidean norm and λ is the tuning parameter that controls the sparsity of the coefficients at the group level. It should be noted that this computation does not provide within-group sparsity, i.e., the coefficients of all the predictors in a group are either zero or non-zero. A range of 100 λ values is used (default), generated as a fraction of $\lambda_{max}$, the smallest λ value for which all the coefficients are zero. The strength of association between a group of predictors and the response variable is determined by the λ value corresponding to the entry of that group into the regression equation, with higher λ value corresponding to a stronger association. The group lasso model was run for 500 iterations (stability selection) for robustness. The statistical significance of subnetworks obtained from NetGSA was not taken into account while performing group lasso regression.

### 3.5. Data and Resource Availability

Mouse T1D metabolomics data analyzed during the current study are available in the Metabolomics Workbench repository (Study ST000057). Metabolomics data from the Framingham Heart Study Offspring Cohort analyzed during the current study are available on dbGaP (https://www.ncbi.nlm.nih.gov/gap/) with the study accession number phs000007.v29.p10 and dataset phenotypic identifiers ‘pht002234.v5.p10:’ (Metabolomics-HILIC), ‘pht002894.v1.p10:’ (Central Metabolomics-HILIC), ‘pht002343.v4.p10:’ (Metabolomics-Lipid Platform). Lipidomics data from the Michigan Mother-Infant Pairs Cohort (MMIP) analyzed during the current study are available in the Metabolomics Workbench repository (Project ID PR000386). Filigree is freely available at http://metscape.ncibi.org/filigree.html. Scripts associated with the current analyses are available at https://github.com/griyer/Diabetes_manuscript_code.git.

## Figures and Tables

**Table 1:** Number of edges discovered with and without subsampling the Framingham Heart Study Offspring Cohort T2D data.

	Number of Edges		
	Non-Diabetic	Diabetic	Common
Without subsampling	784	73	250
With subsampling	281	36	223

**Table 2:** Summary of the node-aggregation and identified subnetworks in each pairwise comparison of the MMIP lipidomics data.

Comparison	Effective Number of Features	% Reduction in Feature Space	Number of Significant Subnetworks (Adj *p*-Value < 0.05)	Total Number of Subnetworks Identified
M1—M3	298	55.45	14	19
M1—CB	298	55.45	19	20
M3—CB	286	57.25	9	12

## Supplementary Materials

The following are available online at https://www.mdpi.com/2218-1989/10/12/479/s1, Figure S1: Filigree Partial Correlation Networks from T1D mouse model data highlighting all subnetworks, Figure S2: Partial Correlation Networks from Framingham Heart Study Offspring Cohort T2D data highlighting all subnetworks, Figure S3: Complete Partial Correlation Network from Framingham Heart Study Offspring Cohort T2D data, Figure S4: Subnetwork S1 in the Framingham Heart Study Offspring Cohort T2D data highlighting intermediates of various amino acids’ metabolism and the TCA cycle, Figure S5: Branched chain amino acids-containing subnetwork (S11) in the Framingham Heart Study Offspring Cohort T2D data, Figure S6: Top two M3-CB subnetworks that strongly associated with infant birthweight, Figure S7: Top two M1-M3 subnetworks that strongly associated with infant birthweight, Table S1: Filigree analysis of the T1D (progressors and non-progressors) NOD mice metabolomics data, Table S2: DNEA analysis of the Framingham Heart Study Offspring Cohort T2D data, Table S3: M1-CB Filigree analysis from the MMIP lipidomics data, Table S4: M3-CB Filigree analysis from the MMIP lipidomics data, Table S5: M1-M3 Filigree analysis from the MMIP lipidomics data. References [83,84,85,86,87,88,89,90,91] are cited in the supplementary material.
