# Supplementary material for: Application of Differential Network Enrichment Analysis for Deciphering Metabolic Alterations
Source: Metabolites. 2020 Nov 24;10(12):479. doi: 10.3390/metabo10120479 (PMC7761243; doi:10.3390/metabo10120479)
Supplement: Supplementary file 1 [file metabolites-10-00479-s001.zip › supplementary material.docx]

Application of Differential Network Enrichment Analysis for Deciphering Metabolic Alterations

Gayatri R Iyer^1^, Janis Wigginton ^2^ , William Duren ^1,2^, Jennifer L LaBarre ^3^, Marci Brandenburg ^1,4^, Charles Burant ^5^, George Michailidis ^2,6,^* and Alla Karnovsky ^1,^*

^1^ Department of Computational Medicine and Bioinformatics, University of Michigan Medical School, Ann Arbor, MI, USA; griyer@umich.edu (G.R.I.); wld@med.umich.edu (W.D.); mbradenb@umich.edu (M.B.)

^2^ Michigan Regional Comprehensive Metabolomics Resource Core, Ann Arbor, MI, USA; wiggie@med.umich.edu

^3^ Department of Nutritional Sciences, University of Michigan School of Public Health, Ann Arbor, MI, USA; jenlab@med.umich.edu

^4^ Taubman Health Sciences Library, University of Michigan, Ann Arbor, MI, USA

^5^ Department of Internal Medicine, University of Michigan Medical School, Ann Arbor, MI, USA; burantc@med.umich.edu

^6^ Department of Statistics, University of Florida, Gainesville, FL, USA

***** Correspondence: gmichail@ufl.edu (G.M.); akarnovs@med.umich.edu (A.K.); Tel.: +1-352-294-3913 (G.M.); +1-734-615-9314 (A.K.)

Supplemental Methods

DNEA algorithm

The DNEA methodology has been previously published [21]. The algorithm has three main steps and is described in detail below.

Step 1: Joint estimation of the partial correlation network (PCN) across two groups of samples

Partial correlations measure the conditional dependence between a pair of variables, given all the other variables and for normally distributed data correspond to the non-zero elements of the empirical inverse-covariance matrix (precision matrix). In a high-dimensional setting, where the number of variables (e.g. metabolites) exceeds the number of available samples (*p >> n*), a regularized (penalized) estimate of the precision matrix is needed. Under the assumption that the underlying PCN is relatively sparse the graphical lasso (glasso) algorithm [83] can be employed to estimate the inverse-covariance matrix. Glasso uses *l*_1_-regularization to induce sparsity in the matrix. For an *n x p* data matrix consisting of *n* observations and *p* variables (metabolites), assumed to be distributed according to a multivariate Gaussian distribution with covariance Σ, the precision matrix (inverse-covariance matrix) is given by Θ = Σ^−1^, where the non-zero elements of Θ denote the partial correlation between a pair of variables. Zero entries in Θ indicate conditional independence between the specific pair of variables. The PCN estimate is computed by minimizing the penalized negative log-likelihood function (glasso estimator):

| 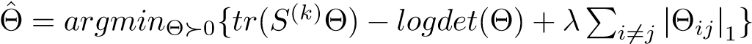 | (1) |
| --- | --- |

where *tr* denotes the trace of the matrix, *det* denotes the determinant of the matrix, and *S* is the sample covariance matrix for condition *k*, given by
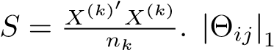
 is the *l*_1_-norm (sum of the absolute values of the elements) of the precision matrix that induces sparsity in the PCN, given that the number of true associations between metabolites is relatively low [84]. *λ* is the regularization parameter that controls the level of sparsity in the network and is selected by minimizing the Bayesian Information Criterion [46].

The first component of the DNEA methodology leverages the glasso algorithm to estimate two related PCNs, by borrowing statistical strength from samples of both groups when estimating similar elements of their corresponding precision matrices. Specifically, given an *n x p* data matrix consisting of *n* observations and *p* variables (metabolites), let *n*_1_ be the number of observations in *group* 1 and *n*_2_ (or *n* − *n*_1_) be the number of observations in *group* 2. For group *k* (where *k* = 1,2), *X*^(^*^k^*^)^ denotes the *n_k_ x p* data matrix. The data are assumed to be distributed according to multivariate Gaussian distributions with covariance Σ^(^*^k^*^)^, with the corresponding precision matrix (inverse-covariance matrix) given by Θ^(^*^k^*^)^ = (Σ^(^*^k^*^)^)^−1^. Further, it is assumed that Θ_1_ ≈ Θ_2_, namely that the presence or absence of edges tends to be relatively conserved across biological/disease states [25,85–87]. Then, DNEA employs a Joint Estimation method (JEM) operationalized by the following optimization problem for *K* groups:

| 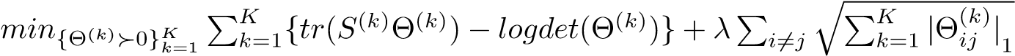 | (2) |
| --- | --- |

The modified penalty term now regularizes edges in the PCNs
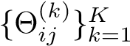
 as a *group* aiming to identify shared attributes. This approach makes use of the entire available sample set and often identifies more edges than might be discovered by analyzing samples of each group separately. Further, in order to recover robust and stable edges in the PCNs, a stability selection [88] procedure is applied to generate selection probabilities for edges in the network. Stability selection involves repeated random subsampling of the data followed by re-fitting the model at each iteration. The edges in the PCNs that are estimated most frequently are retained for downstream analysis.

Step 2: Clustering (unsupervised) of the resulting PCN using consensus clustering

With stable partial correlation networks computed, the next component in the DNEA methodology is the identification of communities or subnetworks consisting of densely interconnected metabolites. DNEA employs a consensus clustering [89] approach to cluster the graph representation of the PCN. Different community detection algorithms may partition the network slightly differently and the use of consensus clustering allows employing multiple clustering algorithms for identifying stable and reliable subnetworks. Briefly, *M* clustering algorithms are applied to the PCNs to yield *M* partitions/clusters. A *p x p* consensus matrix is generated where the *ij-th* entry is the fraction of the clustering algorithms that assigned nodes *i* and *j* to the same subnetwork. The consensus matrix is dichotomized (typically, values less than 0.5 are set to zero) and clustered using the same *M* clustering algorithms to yield a new consensus matrix. These steps are repeated iteratively until the consensus matrix becomes a block diagonal one, which provides the final assignments for the subnetworks.

Step 3: Enrichment analysis of subnetworks with NetGSA algorithm

The final component in the DNEA methodology is testing for enrichment of subnetworks identified by the previous step. This is accomplished by employing the NetGSA algorithm [22,91]. NetGSA (Network-based Gene Set Analysis), originally developed for gene expression data, performs an enrichment analysis that accounts for both differences in the expression level of biomolecules and changes in network topology by using a latent variable model to compute an influence matrix encoding changes in network structure.

Supplementary Results

Comparison of Filigree to existing methods

Filigree combines partial correlation network construction with enrichment testing. To the best of our knowledge, there is currently no pipeline or tool available that performs comparable analyses. Therefore, we decided to compare the network construction and enrichment testing modules of Filigree individually with existing methods that perform similar tasks.

First, we constructed partial correlation networks from the data derived from the type 1 Diabetes (T1D) mice using Gaussian Graphical Model (GGM) method. The computation of a partial correlation network requires the sample size to be at least as large as the number of features. To reduce the feature space, we fit an O-PLS-DA model to the data and selected the top 25 metabolic discriminants differentiating T1D from non-T1D. It must be noted that this approach has limitations as PLS-DA identifies the most discriminant linear combinations of features, selecting the metabolites based on their weights (loadings) is an ad-hoc approach that can leave out metabolites potentially carrying important biological information.

We used these 25 metabolites to build partial correlation networks on the T1D (*n* = 30) and non-T1D (*n* = 41) sample groups separately. The results are summarized below:

**Table 1.** Filigree analysis of the T1D (progressors and non-progressors) NOD mice metabolomics data.

| **Adjusted *p*-Value** | **Number of Edges in Non-T1D Group** | **Number of Edges in T1D Group** |
| --- | --- | --- |
| <0.05 | 2 | 0 |
| <0.1 | 2 | 0 |
| <0.2 | 4 | 0 |
| <0.5 | 10 | 0 |
| <0.9 | 39 | 0 |

Additionally, we selected the top 50 metabolic discriminants from the O-PLS-DA model to build partial correlation network from the entire dataset (*n* = 71). This is similar to the analysis performed by the authors of the original paper [75]. Note that this does not provide any information on the differential status of the edges (partial correlations). Results are summarized below:

**Table 2.** DNEA analysis of the Framingham Heart Study Offspring Cohort T2D data.

| **Adjusted *p*-Value** | **Number of Edges** |
| --- | --- |
| <0.05 | 8 |
| <0.1 | 8 |
| <0.2 | 11 |
| <0.5 | 23 |
| <0.9 | 75 |

As can be seen from the tables, it is not feasible to retrieve sufficient statistically significant partial correlations from individual sample groups, and by combining the experimental groups we lose information on the changes in the network topology accompanying T1D progression.

An alternative to the GGM is the Debiased Sparse Partial Correlation (DSPC) algorithm developed by our group [84] that enables the building of partial correlation networks when the number of features exceeds the number of available samples (*n* < *p*). DSPC is implemented as part of [CorrelationCalculator](http://metscape.ncibi.org/calculator.html) program [84]. We utilized DSPC to construct partial correlation networks with all 163 named metabolites in the T1D dataset, for the non-T1D and T1D groups separately. The following table summarizes results from the non-T1D and T1D samples separately:

**Table 3.** M1-CB Filigree analysis from the MMIP lipidomics data.

| **Adjusted *p*-Value** | **Number of Edges in Non-T1D Group** | **Number of Edges in T1D Group** |
| --- | --- | --- |
| <0.05 | 17 | 6 |
| <0.1 | 22 | 6 |
| <0.2 | 26 | 6 |
| <0.5 | 35 | 8 |
| <0.9 | 39 | 11 |

Consistent with the Filigree analysis, there is a general breakdown of correlations in T1D group. However, the number of statistically significant edges is quite small, limiting the insights that can be derived from this analytical approach. Since networks are constructed separately for each condition, an additional analysis step is required to compare the network topologies.

Alternatively, the network can be constructed using data from both conditions as described above for the GGM approach. The following table summarizes results from the network constructed across all samples (*n* = 71) simultaneously:

**Table 4.** M3-CB Filigree analysis from the MMIP lipidomics data.

| **Adjusted *p*-Value** | **Number of Edges** |
| --- | --- |
| <0.05 | 58 |
| <0.1 | 63 |
| <0.2 | 78 |
| <0.5 | 101 |
| <0.9 | 122 |


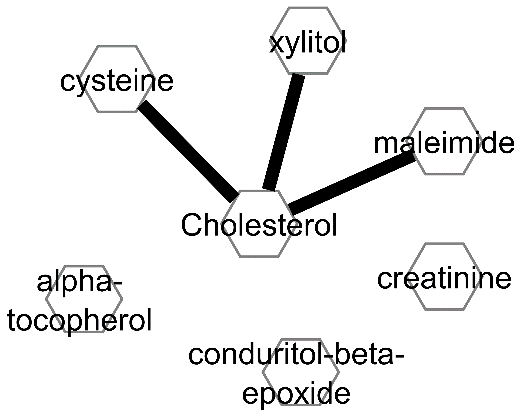


**Figure 1.** Seven metabolites from Subnetwork 1 (Figure 2C of the manuscript) are shown. Filigree identified 9 edges (7 edges in T1D; 2 edges in non-T1D), DSPC identified only 3 significant edges. Moreover, DSPC does not identify differential edges. This especially crucial here as all the edges between these metabolites are differential.

While DSPC analysis generated more edges that passed the significance threshold, it does not provide the additional information on the differential presence of the edges in the two groups. Given a substantial difference in network topology between T1D and non-T1D, this information can be important for understanding the metabolic differences between two groups.

If sample size in not a limiting factor, the above methodology can be used to build partial correlation networks for each condition individually. However, in practice, the number of metabolites often exceeds the number of available samples. In situations like this, Filigree provides greater statistical power by performing joint estimation of the partial correlation networks, while still being able to determine the differential status of the edges between the two conditions, packaged in a single step of analysis.

To compare step 3 of Filigree with existing methods for enrichment testing, we performed Metabolite Set Enrichment Analysis (MSEA) [88] in the T1D mice dataset using Metaboanalyst v4.0 [89]. The following table shows the results from MSEA (FDR < 0.05):

**Table 5.** M1-M3 Filigree analysis from the MMIP lipidomics data.

| **Pathway** | **Total Cmpd** | **Hits** | **Statistic Q** | **Expected Q** | **Raw p** | **Holm p** | **FDR** |
| --- | --- | --- | --- | --- | --- | --- | --- |
| Pantothenate and CoA biosynthesis | 19 | 7 | 8.602 | 1.4286 | 2.89E-06 | 0.000168 | 0.000168 |
| Galactose metabolism | 27 | 10 | 6.0314 | 1.4286 | 3.09E-05 | 0.001762 | 0.000896 |
| Pentose phosphate pathway | 22 | 4 | 10.244 | 1.4286 | 7.93E-05 | 0.004441 | 0.001533 |
| Glycerophospholipid metabolism | 36 | 3 | 8.3451 | 1.4286 | 0.000395 | 0.021733 | 0.00573 |
| Starch and sucrose metabolism | 18 | 7 | 5.7757 | 1.4286 | 0.000844 | 0.045574 | 0.008807 |
| Pentose and glucuronate interconversions | 18 | 3 | 8.2587 | 1.4286 | 0.000911 | 0.048285 | 0.008807 |
| Glycolysis / Gluconeogenesis | 26 | 5 | 5.645 | 1.4286 | 0.001895 | 0.098527 | 0.015699 |
| Amino sugar and nucleotide sugar metabolism | 37 | 5 | 5.4626 | 1.4286 | 0.002625 | 0.13389 | 0.019033 |
| Thiamine metabolism | 7 | 1 | 10.636 | 1.4286 | 0.00551 | 0.27552 | 0.035511 |
| Neomycin, kanamycin and gentamicin biosynthesis | 2 | 2 | 6.5921 | 1.4286 | 0.00838 | 0.41061 | 0.039402 |
| Fructose and mannose metabolism | 20 | 3 | 5.3309 | 1.4286 | 0.008527 | 0.41061 | 0.039402 |
| Steroid biosynthesis | 42 | 1 | 9.5242 | 1.4286 | 0.008832 | 0.41508 | 0.039402 |
| Steroid hormone biosynthesis | 85 | 1 | 9.5242 | 1.4286 | 0.008832 | 0.41508 | 0.039402 |
| Taurine and hypotaurine metabolism | 8 | 2 | 6.5089 | 1.4286 | 0.010161 | 0.45723 | 0.041392 |
| Glycerolipid metabolism | 16 | 3 | 6.1701 | 1.4286 | 0.010705 | 0.47102 | 0.041392 |
| Nicotinate and nicotinamide metabolism | 15 | 3 | 5.2704 | 1.4286 | 0.012231 | 0.52594 | 0.044338 |

In this analysis, most of the significant pathways (FDR < 0.05) are represented by a very small number of metabolites in the data (1–7). Moreover, there are only 44 unique metabolites in these pathways. We also performed Over Representation Analysis (ORA) using 48 metabolites with an FDR < 0.1 in a Student’s t-test and did not find any significant pathways.

The enrichment testing in Filigree utilizes the network-based gene set analysis (NetGSA) algorithm that tests for enrichment not just based on differential metabolite levels but also based on differences in the network topology. Moreover, it does not rely on prior pathway information, thus expanding the scope of its applicability. With well annotated metabolites, one can use the topology from KEGG and NetGSA and get decent results. By building differential networks, we take a more holistic viewpoint and understand where changes come from. Filigree thus provides significant advantage over traditional pathway enrichment approaches.

We chose the T1D mice dataset for the comparative analysis described above because of the well-balanced sample groups. Similar analysis can also be performed with the Framingham Heart Study Offspring Cohort T2D data; however, the highly unbalanced design can hinder the interpretation of the results. We developed the subsampling pipeline in Filigree for exactly this reason. Finally, the Michigan Mother-Infant Pairs (MMIP) Cohort data is a lipidomics dataset and therefore is not amenable to traditional pathway enrichment analysis. Certain tools do exist that perform lipid ontology- or lipid pathway-based enrichment testing (LION and LIPEA respectively). LION [90] ontologies are more focused on physicochemical properties and therefore have limited biological applicability, while LIPEA [91] performs a standard Over-representation analysis (ORA) to identify altered lipid pathways represented in the KEGG database. It therefore does not consider differential levels of lipids between the two conditions. Moreover, lipid metabolism is poorly represented in the KEGG database and majority of the measured lipids do not map to pathways.

In summary, we suggest Filigree provides several advantages for data analysis compared to the existing partial correlation network estimation and enrichment testing methods.

References

1. Friedman, J., Hastie, T. and Tibshirani, R. Sparse inverse covariance estimation with the graphical lasso. *Biostatistics.* **2008**, *9*, 432–441.
2. Gardner, T.S., Di Bernardo, D., Lorenz, D. and Collins, J.J. Inferring genetic networks and identifying compound mode of action via expression profiling. *Science.* **2003**, *301*, 102–105.
3. Jeong, H., Mason, S.P., Barabási, A.L. and Oltvai, Z.N. Lethality and centrality in protein networks. *Nature.* **2001**, *411*, 41–42.
4. Leclerc, R.D. Survival of the sparsest: robust gene networks are parsimonious. *Mol. Syst. Biol.* **2008**, *4*, 213, doi:10.1038/msb.2008.52.
5. Lancichinetti, A. and Fortunato, S. Consensus clustering in complex networks. *Sci. Rep.* **2012**, *2*, 336, https://doi.org/10.1038/srep00336.
6. Xia, J. and Wishart, D.S. MSEA: a web-based tool to identify biologically meaningful patterns in quantitative metabolomic data. *Nucleic Acids Res.* **2010**, *38*, W71–W77.
7. Chong, J., Wishart, D.S. and Xia, J. Using MetaboAnalyst 4.0 for comprehensive and integrative metabolomics data analysis. *Curr. Protoc. Bioinformatics*. **2019**, *68*, e86.
8. Molenaar, M.R., Jeucken, A., Wassenaar, T.A., van de Lest, C.H., Brouwers, J.F. and Helms, J.B. LION/web: a web-based ontology enrichment tool for lipidomic data analysis. *GigaScience*. **2019**, *8*, giz061, doi: 10.1093/gigascience/giz061.
9. Acevedo, A., Durán, C., Ciucci, S., Gerl, M. and Cannistraci, C.V. LIPEA: lipid pathway enrichment analysis. *bioRxiv*. **2018**, 274969, doi: https://doi.org/10.1101/274969.

SUPPLEMENTARY FIGURE

**
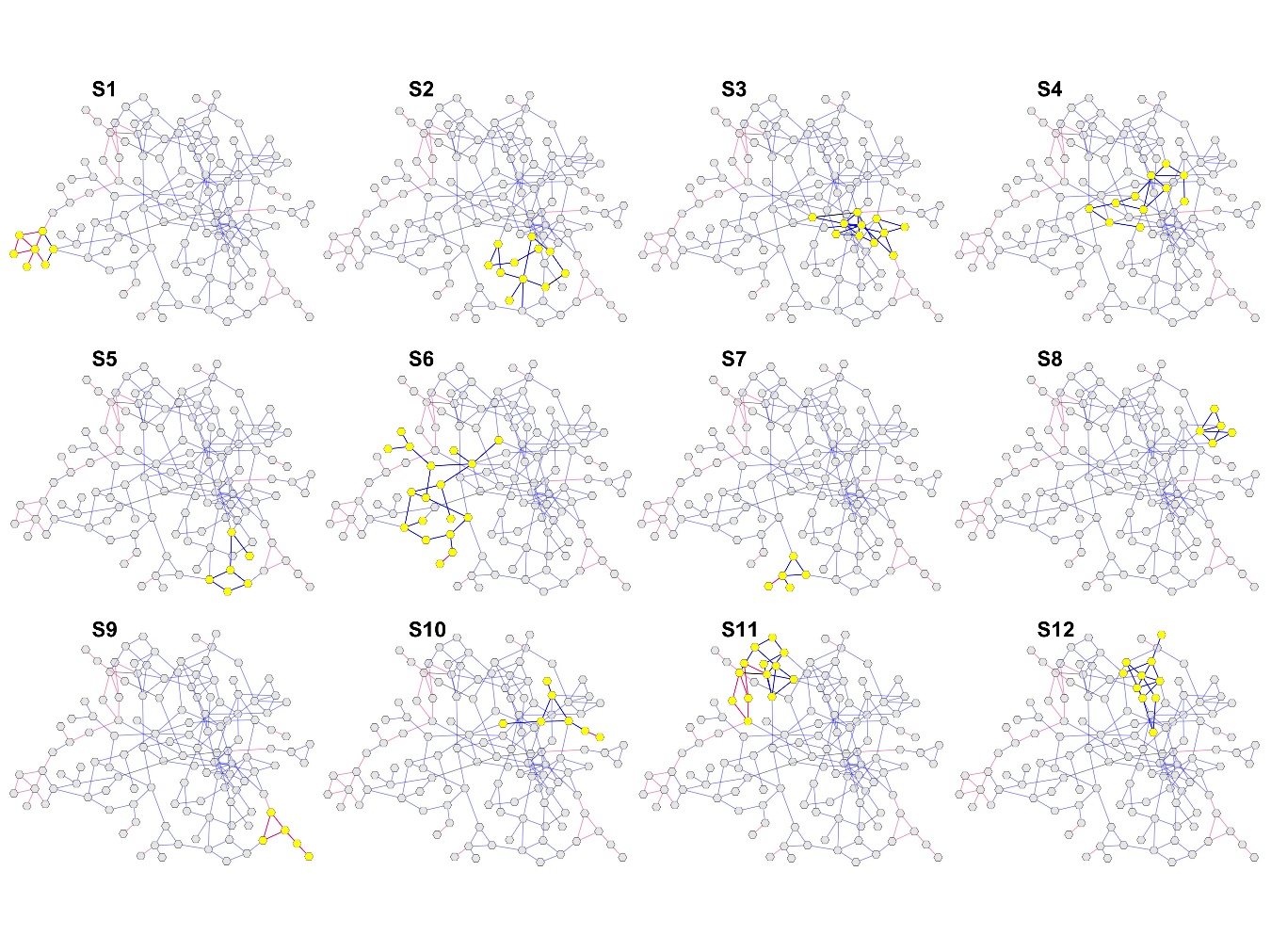
**

**Figure S1.** Complete *Filigree* PCN from T1D mouse model data. Each subnetwork is highlighted in yellow in individual panels. Metabolites with no edges connecting them to this PCN are not shown.


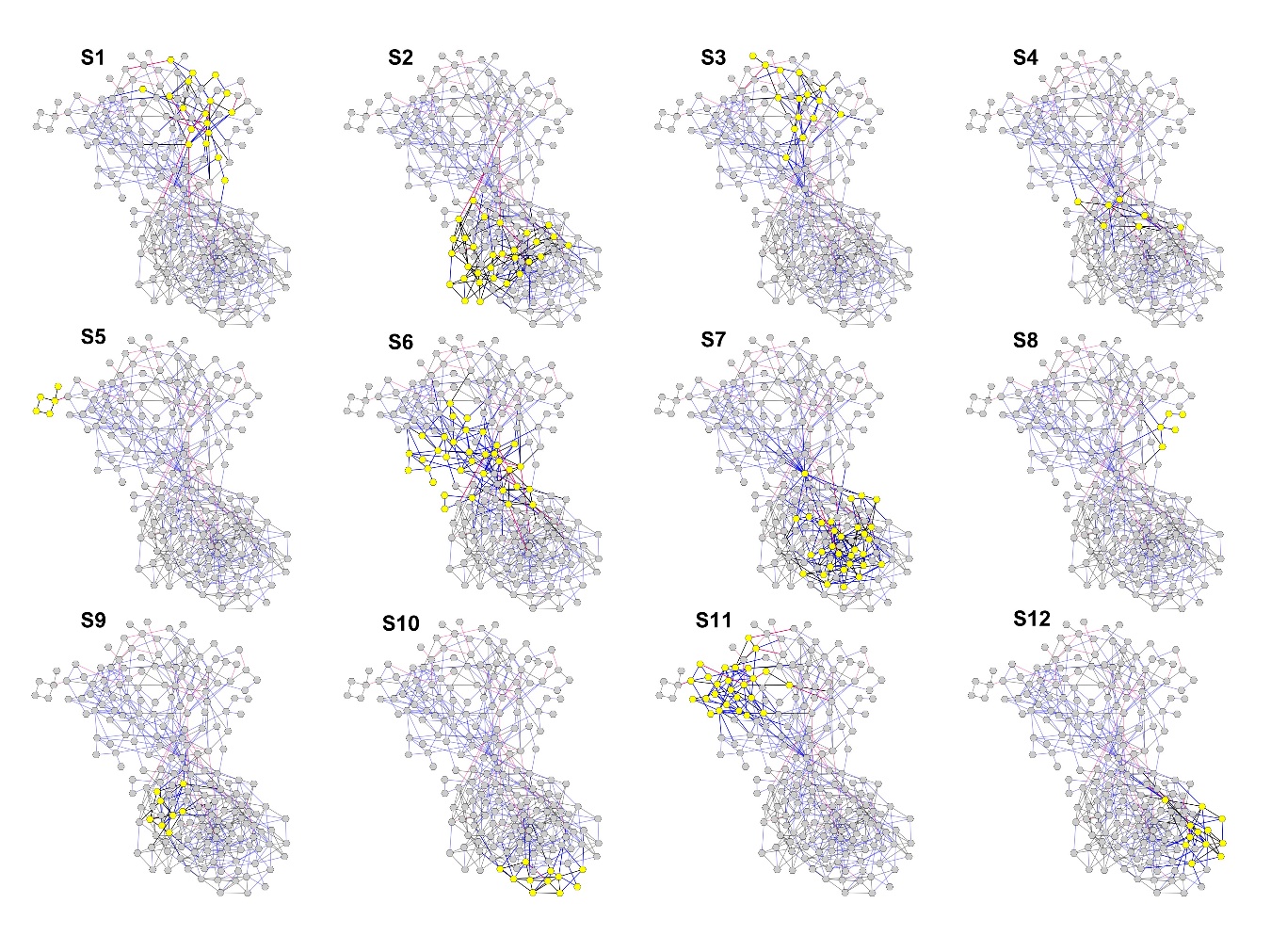


**Figure S2.** Complete PCN from Framingham Heart Study Offspring Cohort T2D data. Each subnetwork is highlighted in yellow in individual panels. Metabolites with no edges connecting them to this PCN are not shown.

**
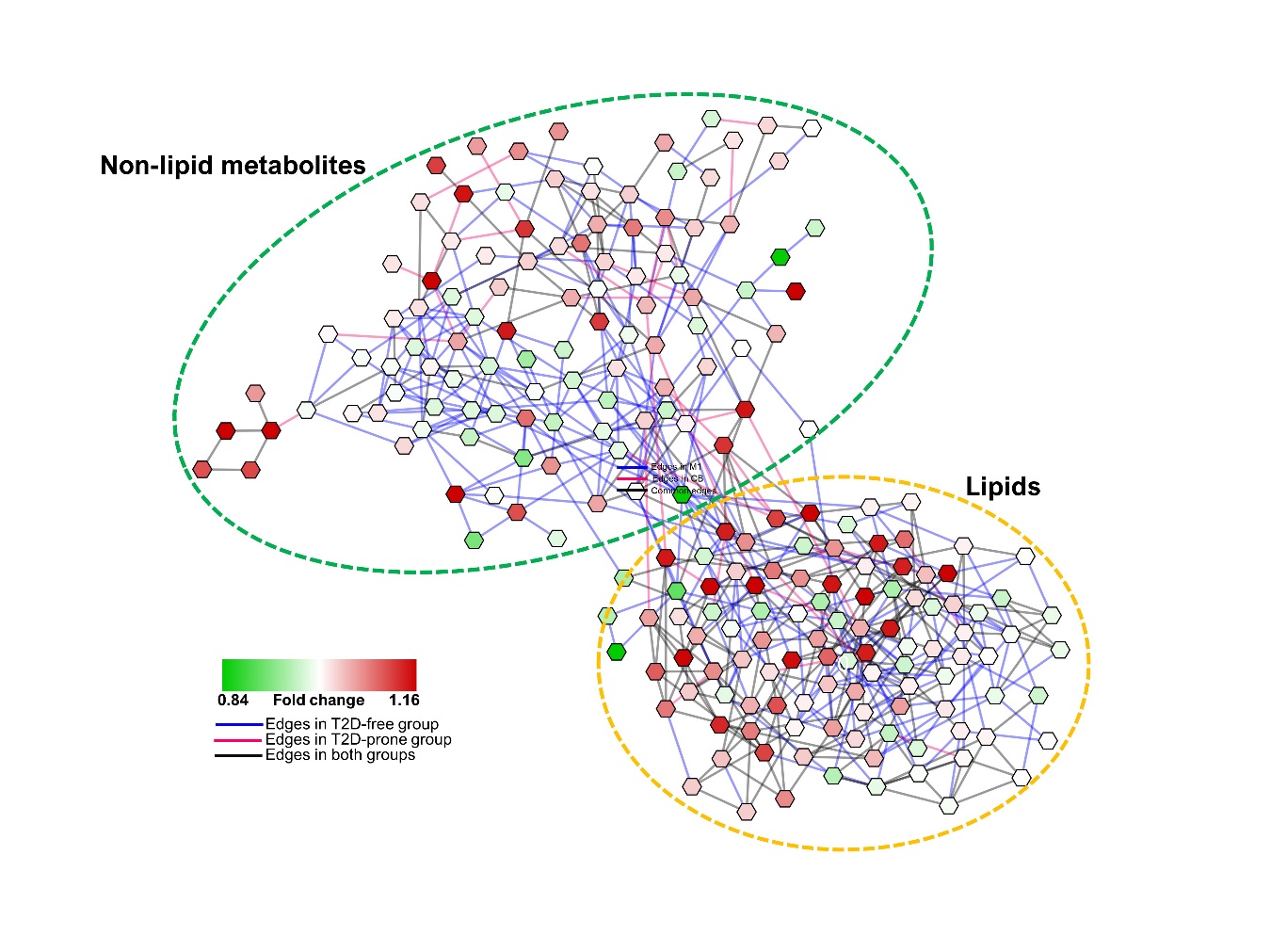
**

**Figure S3.** Complete PCN from Framingham Heart Study Offspring Cohort T2D data. Nodes are colored by fold-change (T2D-prone vs T2D-free) and edges are colored by differential status.


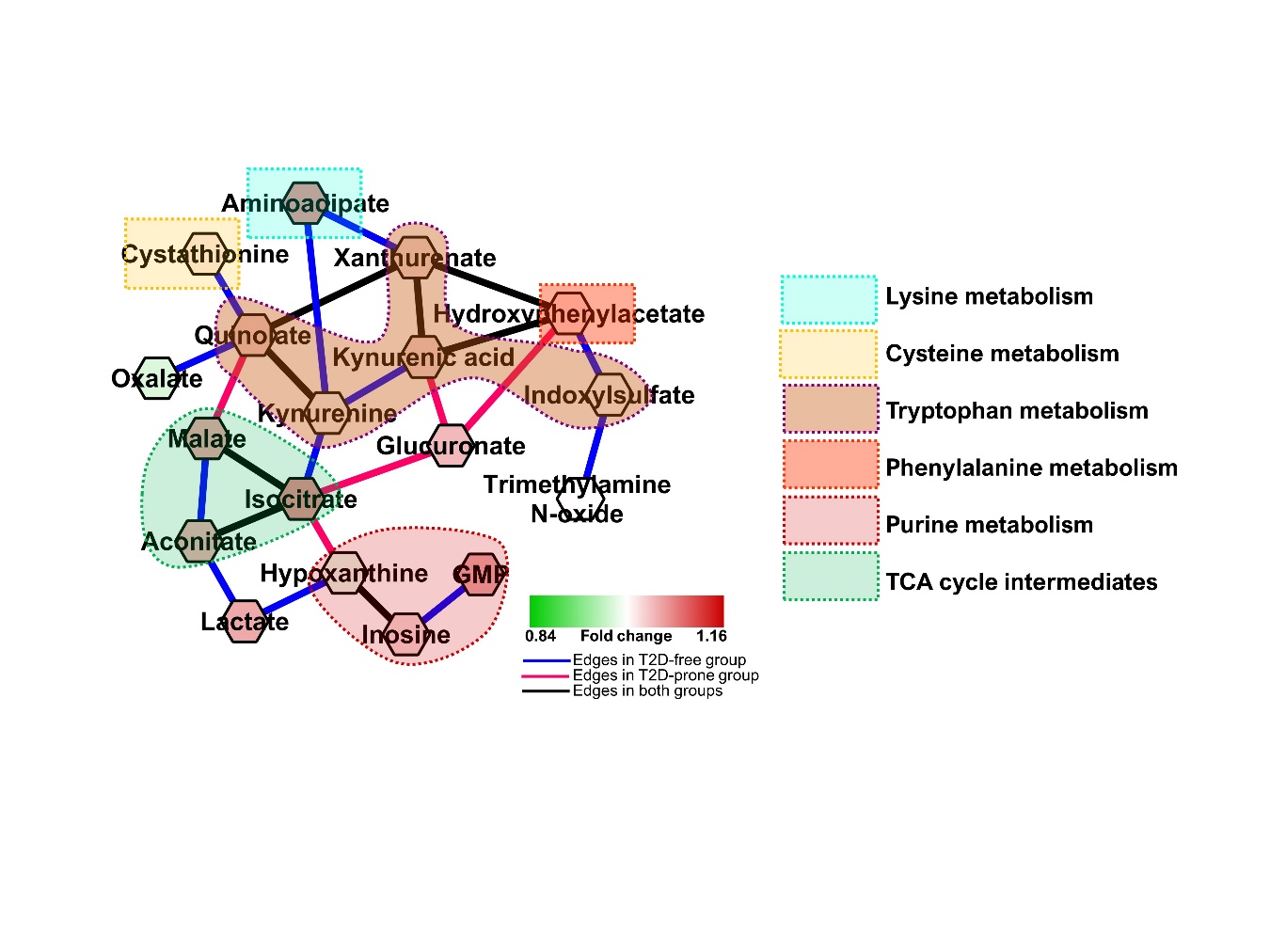


**Figure S4.** Subnetwork S1 in the Framingham Heart Study Offspring Cohort T2D data highlighting intermediates of various amino acids’ metabolism and the TCA cycle.


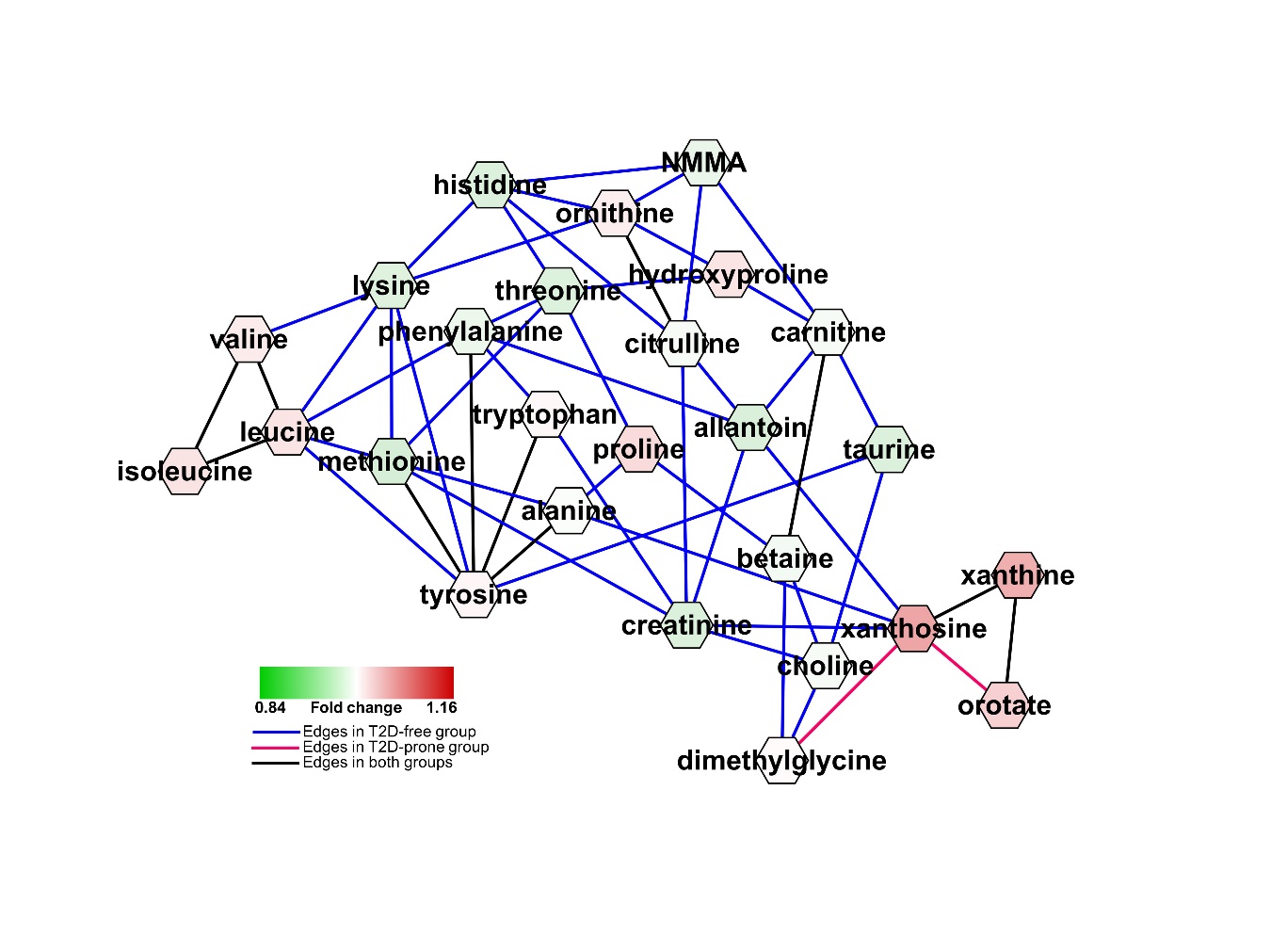


**Figure S5.** BCAA-containing subnetwork (S11) in the Framingham Heart Study Offspring Cohort T2D data.


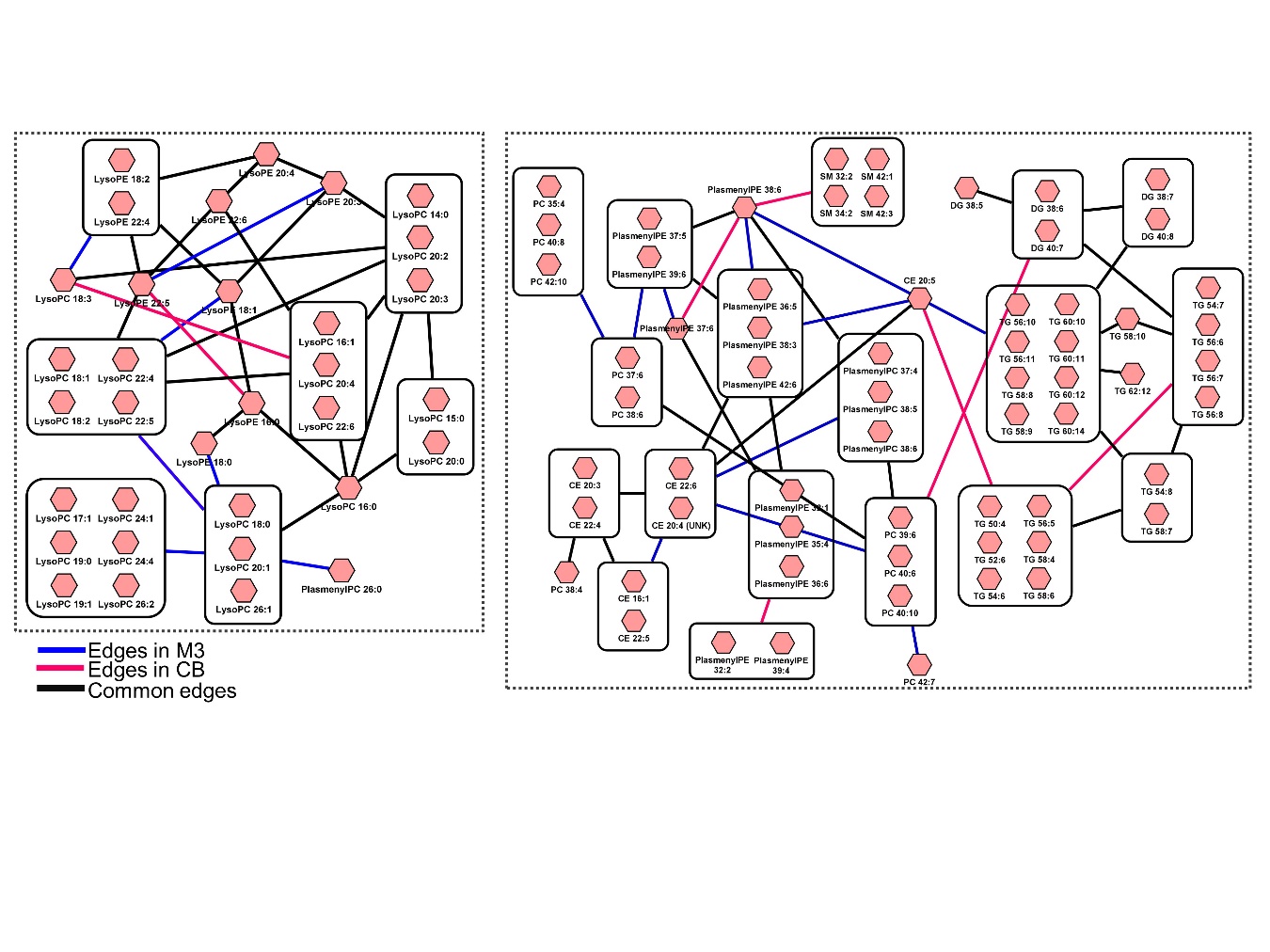


**Figure S6.** Top two M3 vs CB subnetworks strongly associated with infant birthweight. LPC-LPE and CE-PC-PlasmenylPC-PlasmenylPE-DG-TG subnetworks in infant Cord Blood are strongly associated with infant birthweight. Large square nodes containing smaller nodes within them represent ‘aggregated’ nodes with their individual lipid species.


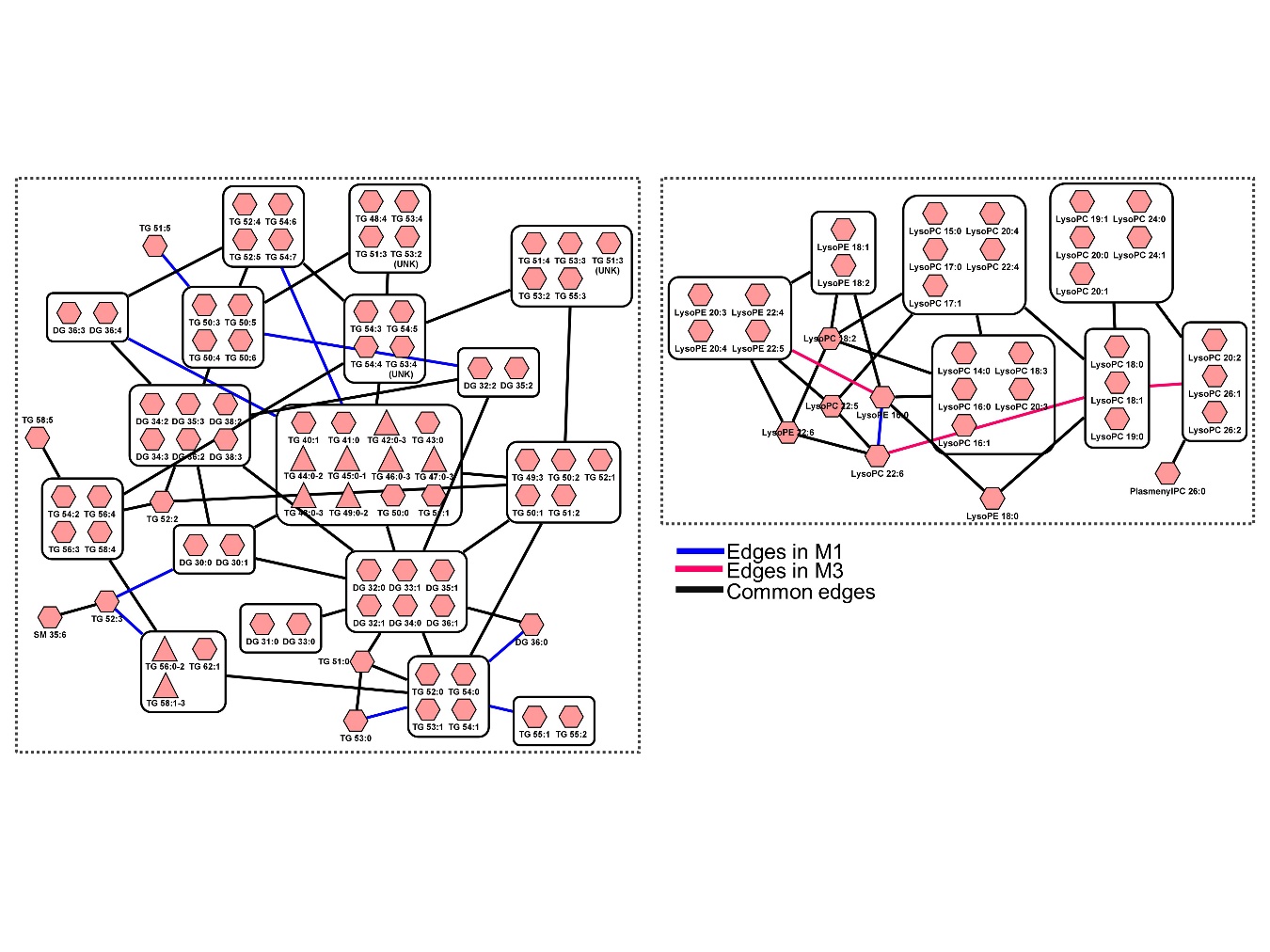


**Figure S7.** Top two M1 vs M3 subnetworks strongly associated with infant birthweight. DG-TG and LPC-LPE subnetworks during the third trimester of the mother are strongly associated with infant birthweight. Large square nodes containing smaller nodes within them represent ‘aggregated’ nodes with their individual lipid species. Triangular nodes represent a small group of triglycerides (2-3) with the same chain length and sequential unsaturation units.
