# Supplementary figures and images for: Application of Differential Network Enrichment Analysis for Deciphering Metabolic Alterations
Source: Metabolites. 2020 Nov 24;10(12):479. doi: 10.3390/metabo10120479 (PMC7761243; doi:10.3390/metabo10120479)

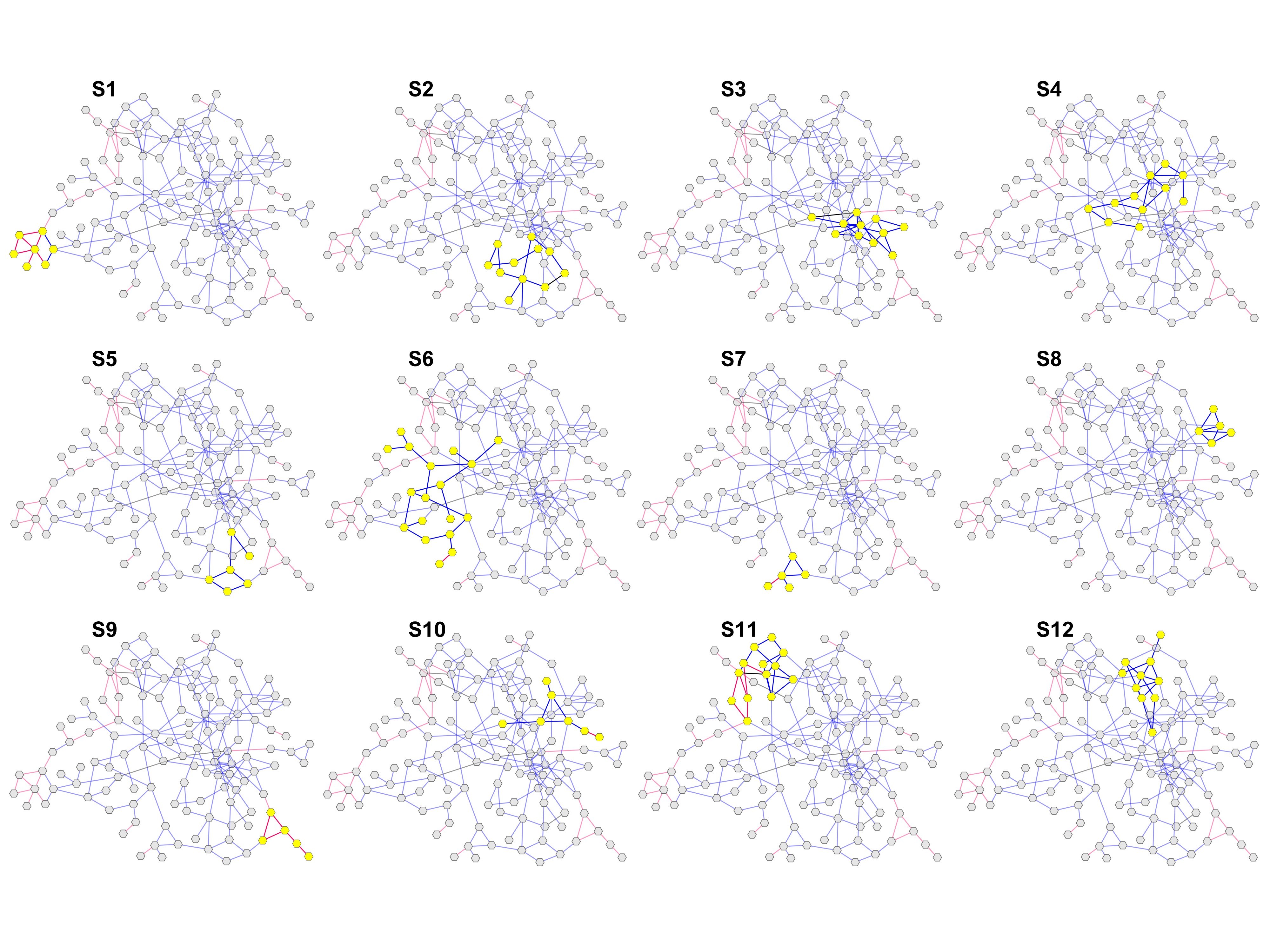

Supplement: Supplementary file 1 [file metabolites-10-00479-s001.zip › FigureS1.jpg]

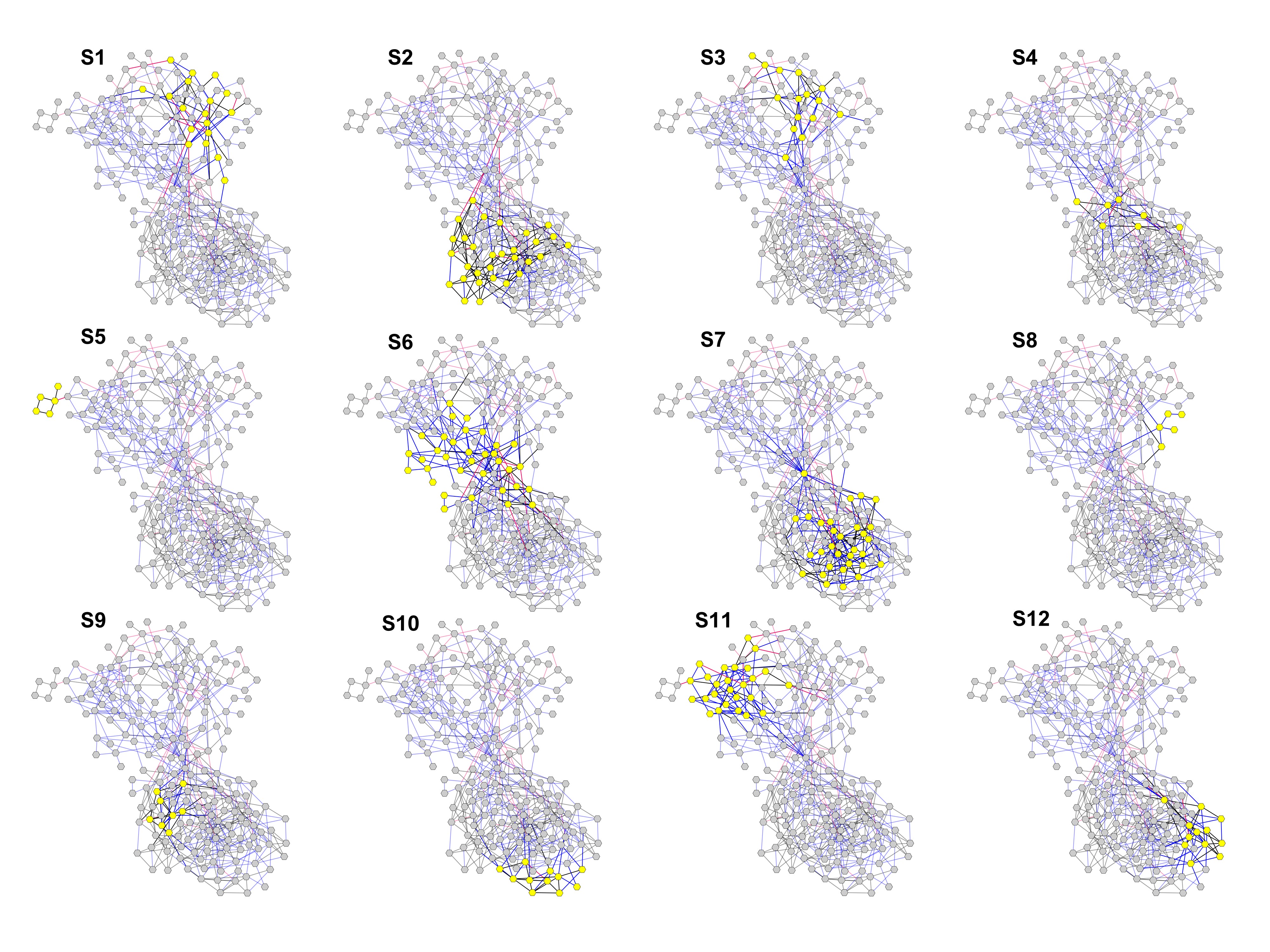

Supplement: Supplementary file 1 [file metabolites-10-00479-s001.zip › FigureS2.jpg]

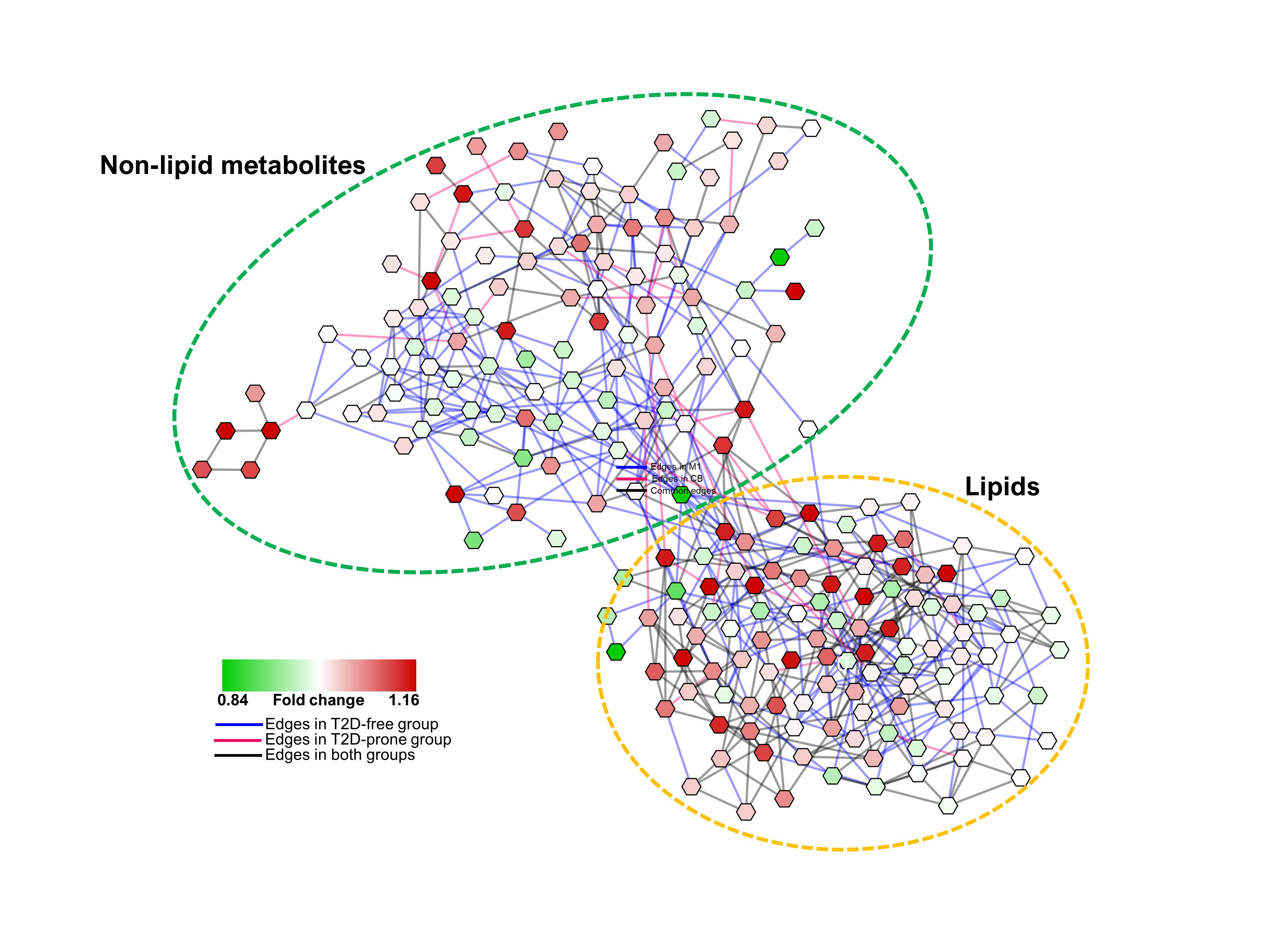

Supplement: Supplementary file 1 [file metabolites-10-00479-s001.zip › FigureS3.jpg]

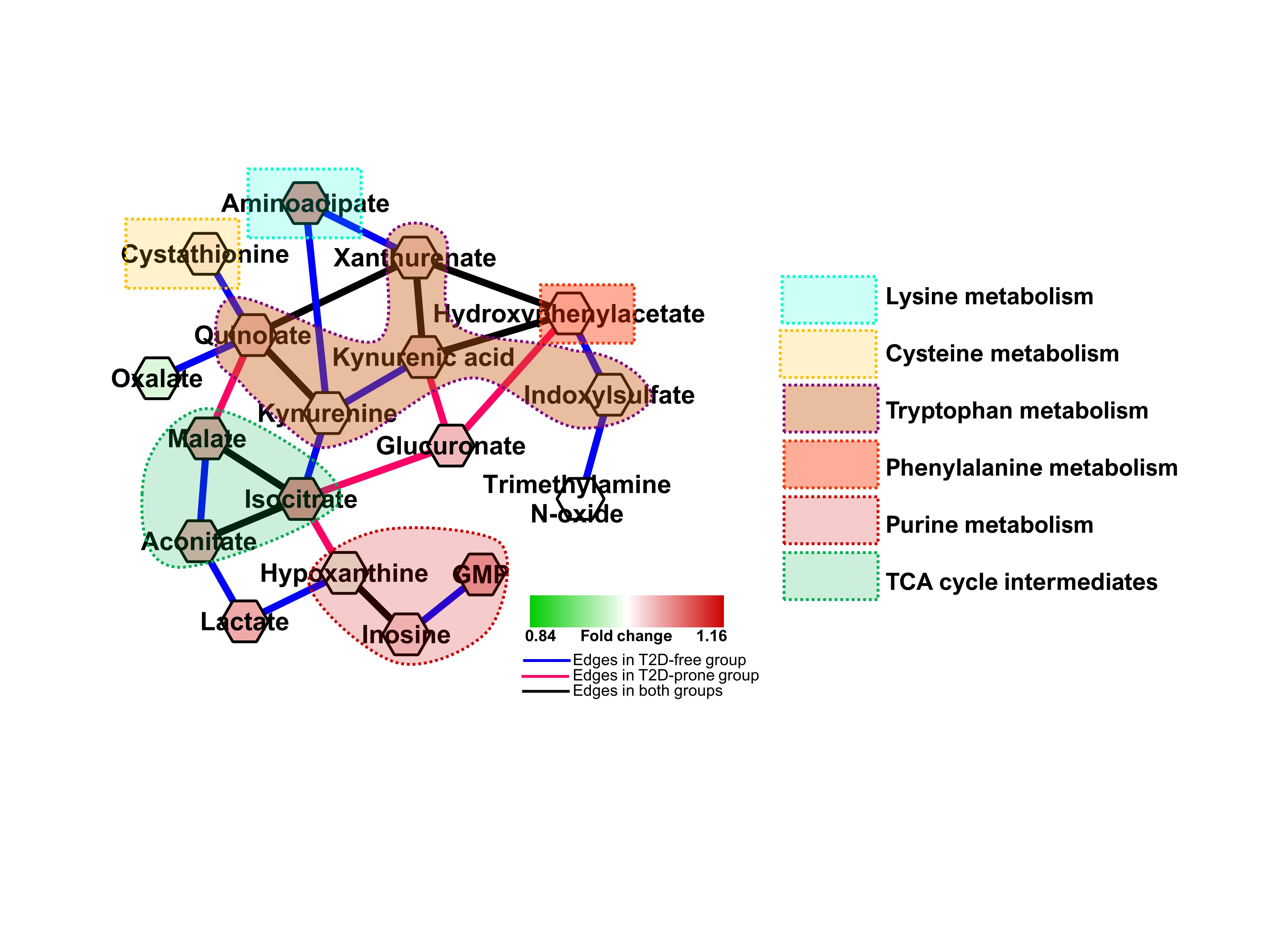

Supplement: Supplementary file 1 [file metabolites-10-00479-s001.zip › FigureS4.jpg]

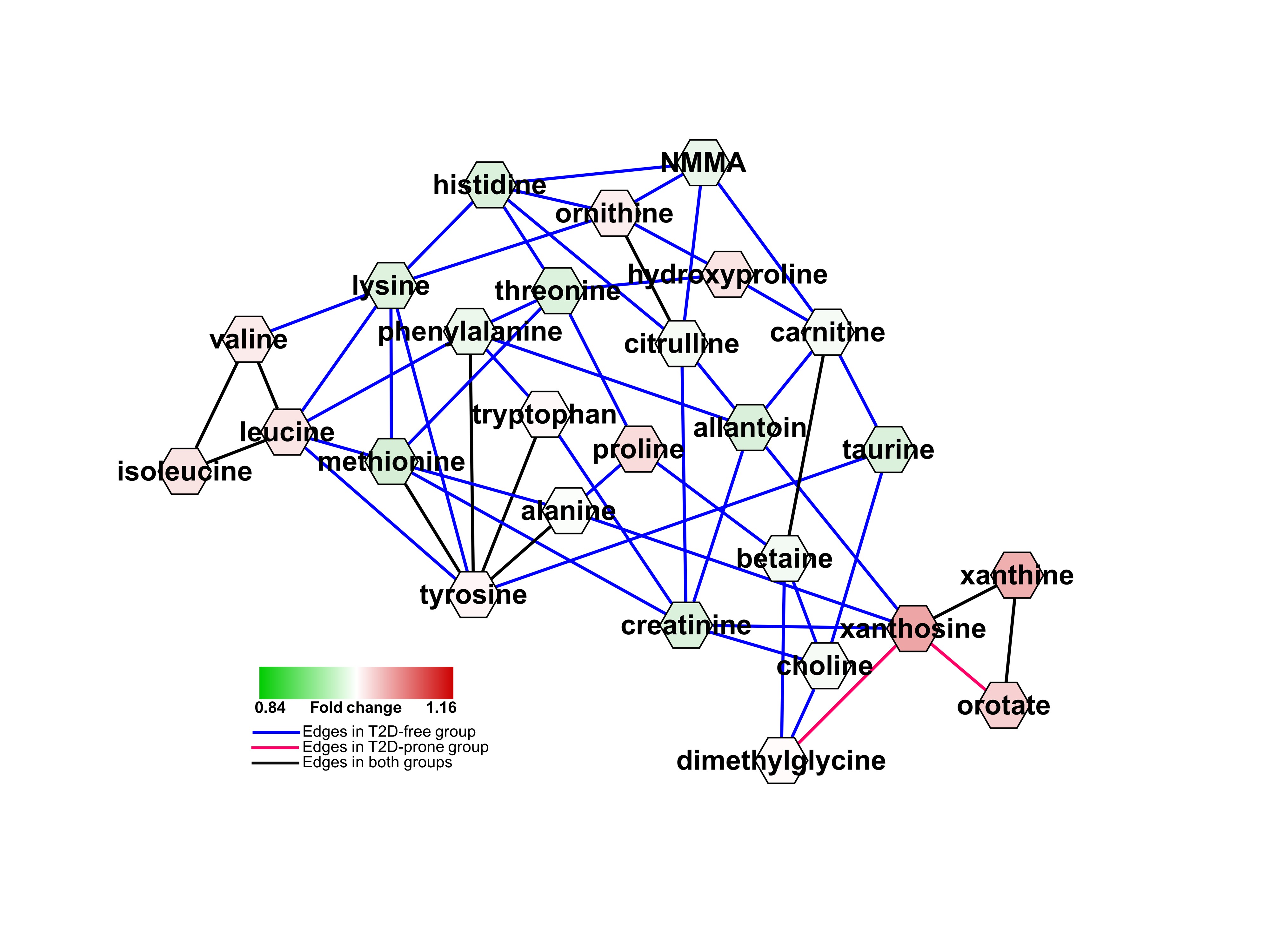

Supplement: Supplementary file 1 [file metabolites-10-00479-s001.zip › FigureS5.jpg]

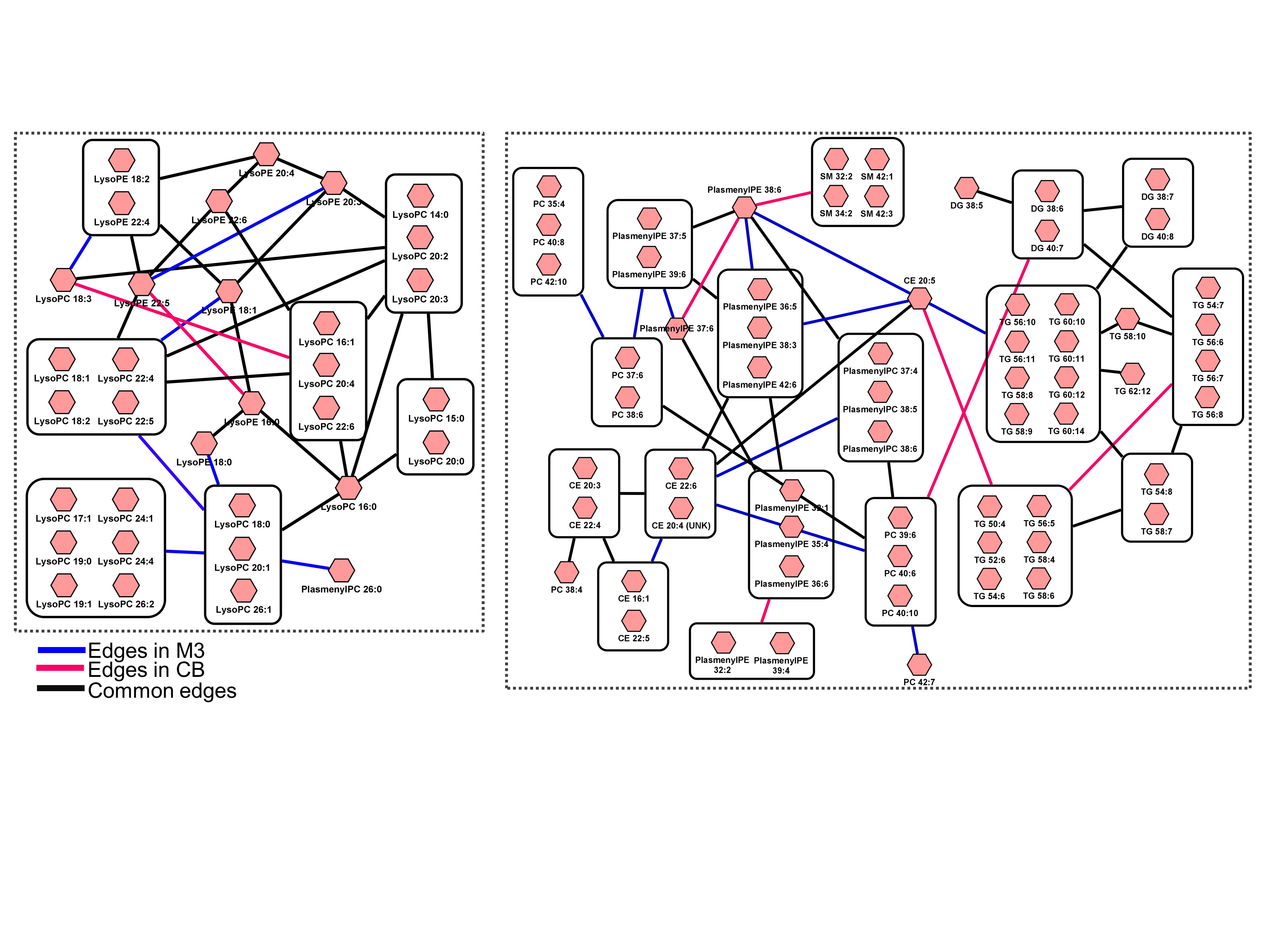

Supplement: Supplementary file 1 [file metabolites-10-00479-s001.zip › FigureS6.jpg]

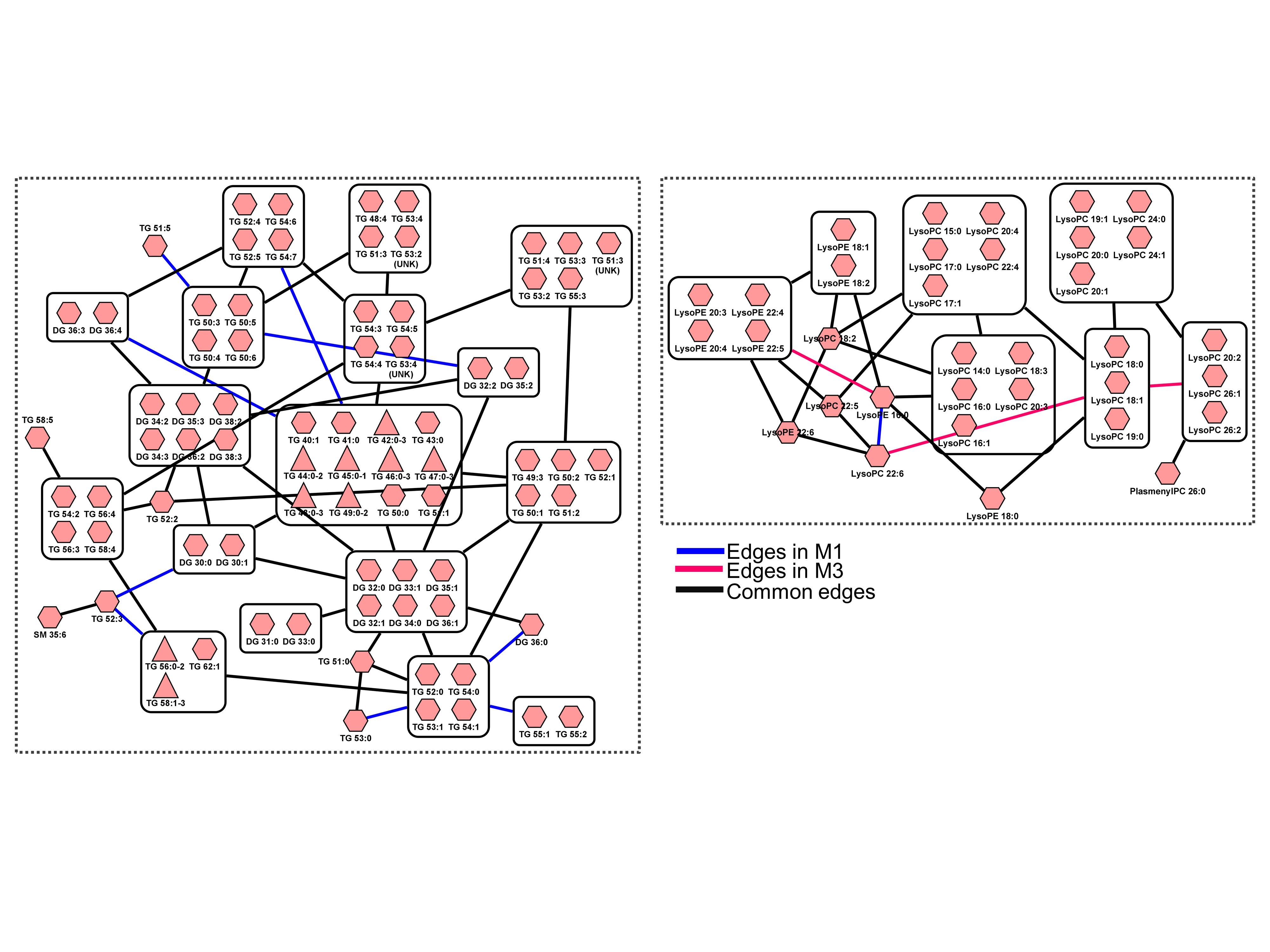

Supplement: Supplementary file 1 [file metabolites-10-00479-s001.zip › FigureS7.jpg]
